# Near-Temperature-Independent Electron Transport Well beyond Expected Quantum Tunneling Range via Bacteriorhodopsin Multilayers

**DOI:** 10.1021/jacs.3c09120

**Published:** 2023-11-07

**Authors:** Sudipta Bera, Jerry A. Fereiro, Shailendra K. Saxena, Domenikos Chryssikos, Koushik Majhi, Tatyana Bendikov, Lior Sepunaru, David Ehre, Marc Tornow, Israel Pecht, Ayelet Vilan, Mordechai Sheves, David Cahen

**Affiliations:** † Department of Molecular Chemistry and Materials Science, Weizmann Institute of Science, Rehovot 7610001, Israel; ‡ Molecular Electronics, Technical University of Munich, 85748 Garching, Germany; § Fraunhofer Institute for Electronic Microsystems and Solid State Technologies (EMFT), 80686 München, Germany; ∥ Department of Chemical Research Support, Weizmann Institute of Science, Rehovot 7610001, Israel; ⊥ Department of Chemistry and Biochemistry, University of California, Santa Barbara, California 93106, United States; # Department of Immunology and Regenerative Biology, Weizmann Institute of Science, Rehovot 7610001, Israel; % Department of Chemical and Biological Physics Weizmann Institute of Science, Rehovot 7610001, Israel; & School of Chemistry, Indian Institute of Science Education and Research, Thiruvananthapuram 695551, Kerala, India; @ Department of Physics and Nanotechnology, College of Engineering and Technology, SRM Institute of Science and Technology, Kattankulathur, Chennai 603203, Tamil Nadu, India

## Abstract

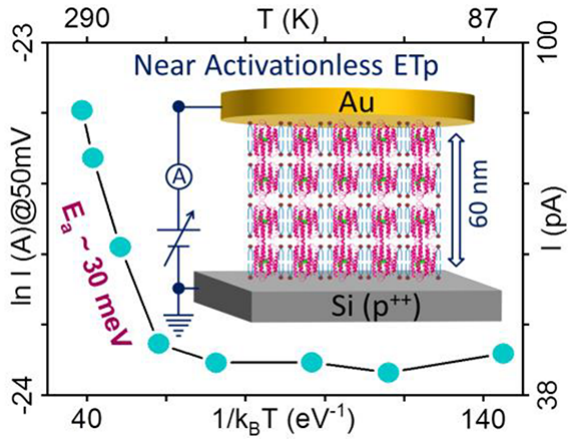

A key conundrum of biomolecular electronics is efficient electron transport (ETp) through solid-state junctions up to 10 nm, often without temperature activation. Such behavior challenges known charge transport mechanisms, especially via nonconjugated molecules such as proteins. Single-step, coherent quantum-mechanical tunneling proposed for ETp across small protein, 2–3 nm wide junctions, but it is problematic for larger proteins. Here we exploit the ability of bacteriorhodopsin (bR), a well-studied, 4–5 nm long membrane protein, to assemble into well-defined single and multiple bilayers, from ∼9 to 60 nm thick, to investigate ETp limits as a function of junction width. To ensure sufficient signal/noise, we use large area (∼10^–3^ cm^2^) Au–protein–Si junctions. Photoemission spectra indicate a wide energy separation between electrode Fermi and the nearest protein-energy levels, as expected for a polymer of mostly saturated components. Junction currents decreased exponentially with increasing junction width, with uniquely low length-decay constants (0.05–0.5 nm^–1^). Remarkably, even for the widest junctions, currents are nearly temperature-independent, completely so below 160 K. While, among other things, the lack of temperature-dependence excludes, hopping as a plausible mechanism, coherent quantum-mechanical tunneling over 60 nm is physically implausible. The results may be understood if ETp is limited by injection into one of the contacts, followed by more efficient charge propagation across the protein. Still, the electrostatics of the protein films further limit the number of charge carriers injected into the protein film. How electron transport across dozens of nanometers of protein layers is more efficient than injection defines a riddle, requiring further study.

## 1 Introduction

Proteins are fascinating candidates for use in biomolecular electronics because of their diverse properties and functions.^1−3^ Previous studies have shown that electronic charge transport, ETp, via protein junctions are influenced by the protein structure,^4^ electrode–protein coupling,^5−7^ and the alignment of frontier molecular orbital energies with respect to the Fermi levels (*E*_F_) of the electrodes.^8−10^ To explore and understand the mechanism(s) of electron transport, we studied junctions of ultrathin protein layers, sandwiched between the two contact electrodes.^11−13^ Such junctions exhibit electronic conductance comparable to or at times even higher than those via conjugated organic molecules.^14^ Still, we expect that the protein layer width would also set a limit on the measurable ETp.

The literature on long-range protein ETp studies is limited, including, for example, on ETp through μm^15^ to cm^16^ long protein fiber or junctions of up to μm thick protein multilayers.^17−19^ Consideration of the distance role in ETp requires verification of the actual transport length of the sandwiched layer between the top and bottom electrodes and specifically the lack of artifacts such as (i) the quality of the protein layer in the junction, (ii) the effect of metal filaments, and (iii) the influence of pinholes. Pinholes are the defective voids in a protein self-assembled layer that allow unwanted contact of the exposed bottom electrode to the top electrode, which leads to electrical shorts, or sometimes partial shorts, with an unstable *I*–*V* response with higher characteristic junction current than expected for a pinhole-free junction. After a few voltage sweeps, this ultimately leads to an electrically shorted junction.

All of these require rigorous care and controlled experiments. We chose bacteriorhodopsin (bR), a thermally stable membrane protein that forms vesicles upon treating with a detergent, octylthioglucoside (OTG), and upon deposition on Si/SiO_*x*_ and Au substrates and subsequent drying (see below) yields good quality bR bilayer films.

Bacteriorhodopsin (bR) functions as a light-driven proton pump in the purple membrane of*Halobacterium salinarum*.^20^ bR is composed of three homomonomers, each constructed of two antiparallel *β*-sheets and seven near parallel transmembrane helices. It contains a light-sensitive retinal chromophore, covalently bound at the central region of the protein via a protonated Schiff-base to a lysine residue (PDB 1FBB).^21,22^ The presence of the relatively electron-rich retinal and the high content of aromatic amino acids (∼13%; 31 per monomer) make it an interesting protein for ETp measurements. bR is a transmembrane protein embedded in the lipid matrix.^22^ Its polar parts, with net negative surface charges at experimental pH 6.4, are exposed to the cytoplasmic and extracellular environments at the membrane’s inner and outer surfaces. In the polar, hydrophilic parts, there are several acidic and basic amino acid residues, which are solvent-accessible and responsible for building up the surface charge. This charge allows electrostatic surface immobilization and also makes these parts potential sites for inter-protein covalent cross-linking by amide bond formation. A pure bR suspension forms a nonuniform, inhomogeneous layer of protein patches on a solid substrate.^23^ However, treatment with OTG yields doughnut-like bR vesicles with a narrow size distribution.^24^ During purification, OTG is removed from the bR vesicles by dialysis,^24^ leaving bR trimers embedded in part of the native lipid. Such vesicle structure favors the formation of good quality, uniform protein bilayers (i.e., with double lipid bilayers) over large substrate areas. Such films are suitable for forming junctions with ∼10^–3^ cm^2^ geometrical area. All characterizations and electrical measurements are consistent with the formation of reproducible, uniform films.^25,26^ The relatively large geometric area allows us to measure small current densities. This is possible even if the electrically active over geometric area ratio, estimated to be up to 10^–4^ in the junctions we use,^11^ is considered (as it will still be equivalent to a 100% area-efficient 3 × 3 μm^2^ junction). All current densities reported here are per geometric area.

Impressively high ETp rates were observed via bR bilayers (originally assumed to be monolayers),^26^ comparable to those measured across ∼4–5× thinner protein junctions, formed with small proteins such as BSA, Cyt C, or Az.^14,27^ Hence, bR is an efficient electron transport medium with a small distance decay constant (*β*, see eq 1).^28^ Earlier, we^25−27,29−31^ and others^32^ studied and reported on junctions made with single bR bilayers as well as with delipidated bR monolayers.^31^

The primary goal of this study is to investigate if such efficient ETp persists also through thicker junctions produced by forming bR multiple bilayers. In the limit of small transmission through a barrier, the tunneling current, *I*, decays exponentially with distance, *r*:
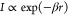
1where *β* is known as the tunneling decay coefficient. Reported *β* values for proteins vary between 1 and 3 nm^–1^,^14^ implying that each nm increase in thickness attenuates the current by 3–20×. Therefore, we expect that ETp across such increasingly thicker protein junctions (few 10s of nm) should decrease to a signal that is not discernible anymore from the noise.

From here onward, we use *β* as a phenomenological parameter, except where noted otherwise. We find a weak length-dependence of ETp via the bR bilayer junctions (*β* ≤ 0.5 nm^–1^), which decreases to (*β* ∼ 0.1 nm^–1^) toward the 60 nm extreme of the widths we have measured. The mere fact that we can measure current via a 60 nm film at 50 mV bias (∼30 nA/cm^2^) is remarkable. Furthermore, the ETp was found to be close to activationless in measurements from room temperature to ∼160 K and completely constant at lower temperatures. The longest distance tested here (60 nm) is at least an order of magnitude longer than the threshold length for transition in ETp mechanism (tunneling to hopping) observed for junctions of rigid, all-conjugated organic molecules.^33,34^ Still, the temperature-independent *I*–*V* response at low applied bias, taken together with the UPS-derived protein HOMO (ionization energy), relative to the *E*_F_ of the contacts and the small normalized differential conductance (NDC) values with mild voltage dependence, is apparently consistent with the operation of direct tunneling. However, direct tunneling is incompatible with the quantum physical limits of the wave function extension in an organic molecular medium without long-range periodicity and with only some isolated conjugation/aromaticity, such as proteins.

## 2 Experimental Section

### 2.1 Protein Solution Preparation

**Figure 1:**
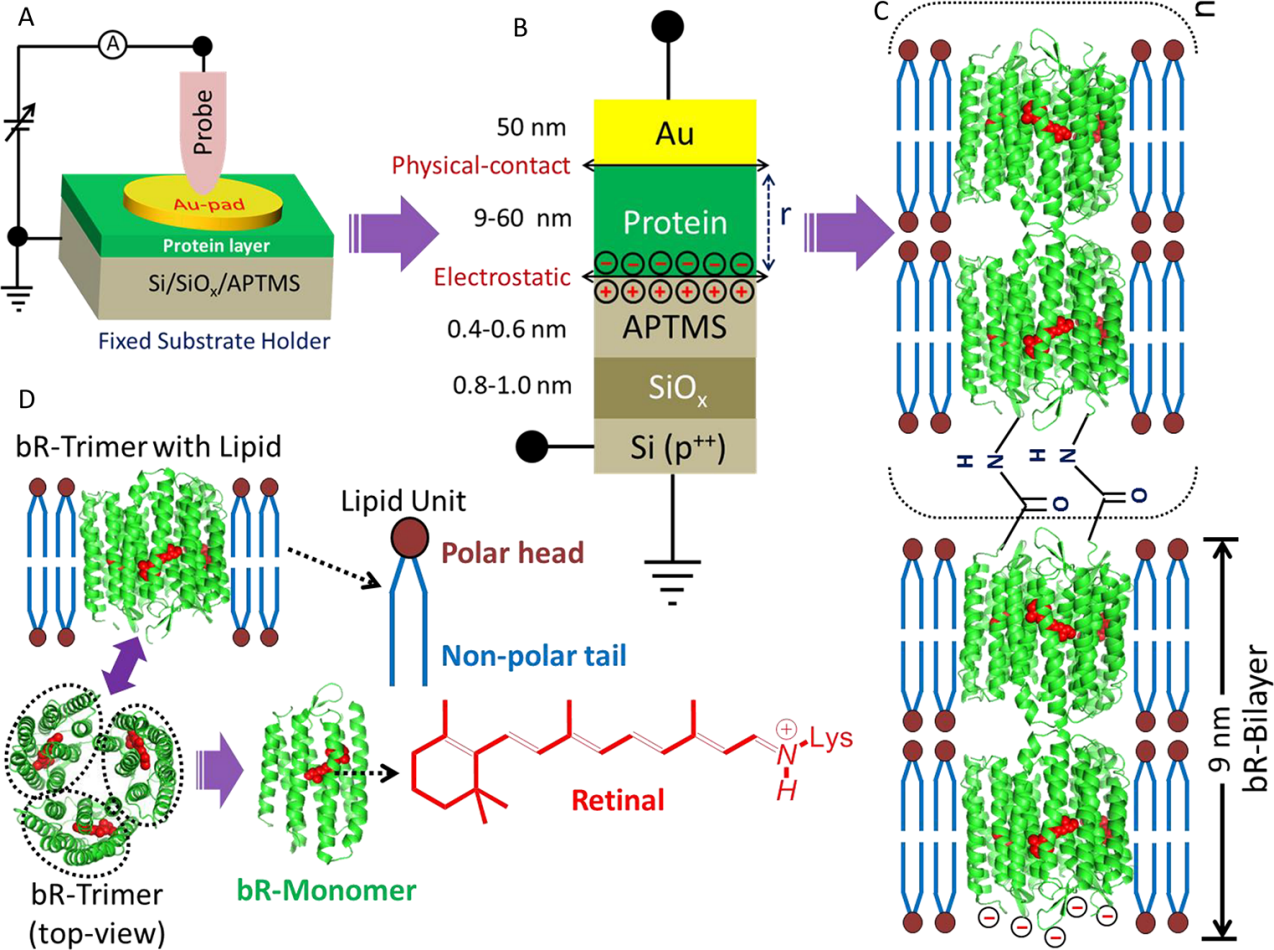
(A) Scheme of the protein junction and electrical circuitry, used for ETp measurements. (B) Scheme of the junctions’ cross section with protein multilayer (thickness; *r*). (C) Tentative scheme of the protein film, consisting of multiple bR bilayers; *n* = 0, 1, 2. The bilayers are covalently cross-linked upon reaction with EDC; each layer consists of the protein part, including retinal, and the associated native lipids. (D) Individual components of protein layers: bottom-left (green) is the protein bacteriorhodopsin, bR [PDB 1BRR (trimer) and 1FBB (monomer)] in its homotrimeric form, as can be seen in the top view (bottom-left corner), where each monomer of the trimer is surrounded by a dotted ellipse, with its retinal chromophore (in red) inside. The retinal chemical structure is shown in bottom-middle (red). bR is a membrane protein situated within a matrix of native lipids (middle, blue).

The bR vesicles were prepared from the purple membranes isolated from *Halobium salinarum* by octylthioglucoside (OTG) treatment as previously described.^22,25^ The bR was stored in 10 mM phosphate buffer (PB), with 0.1 M ammonium sulfate ((NH_4_)_2_SO_4_) at pH 6.4 at room temperature. This solution was buffer diluted to 4 μM for the on-surface immobilization of bR protein molecules. For brevity, all further references to “bR” means final OTG-free,^22^ part of the native lipid–protein complex layers (see Figure 1).

### 2.2 Preparation of Protein Layers

#### 2.2.1 Substrate Cleaning

We used both silicon and gold as substrates for the protein layers. All experiments were done using Si wafers as substrate and electrode, except for PMIRRAS and, in part, UPS measurements, where Au was used. Si substrates were heavily B-doped p^++^-Si (100) with ρ ∼ 0.75 mΩ·cm (from Virginia Semiconductor, USA). Au substrates were prepared by evaporating 45 nm of Au on 5 nm of Cr on solvent-cleaned (acetone, 2-propanol) lightly (P) doped Si (100). The p^++^-Si substrates were solvent-cleaned by 5 min bath sonication in the following solvents, in sequential order; acetone, 2-propanol, and Milli-Q water, followed by N_2_ drying and O_2_:Ar (=1:1) plasma etching for 5 min. Then, the substrate was immersed in 2% HF for 2 min to obtain H-terminated Si, rigorously washed with ultrapure deionized water (Milli-Q), and immersed in hot (80 °C) acid piranha (H_2_SO_4_:H_2_O_2_ = 2:1 by v/v) for 2–3 s for the controlled growth of <1 nm SiO_*x*_. Immediately after the piranha treatment, the substrate was washed with excess Milli-Q water, followed by bath sonication in Milli-Q water for 1 min. Finally, water rinsing and N_2_ drying made the substrate ready for further steps.

The Au substrate was solvent-cleaned similar to the p^++^-Si substrate. Subsequently, after cleaning with solvents, the Au substrate was treated with the hot (80 °C) base piranha (H_2_O:H_2_O_2_:NH_3_ = 5:1:1 by v/v) for 30–40 s, followed by thorough Milli-Q water washing, 1 min bath sonication in Milli-Q water, and N_2_ drying.

bR lacks cysteine residue available for direct binding to a Au substrate; instead, the net negative surface charge of bR in the experimental pH range (6–7) has been used for electrostatic protein binding to a positively charged linker molecules bound to the substrate because neither substrates are positively charged by nature (Au is neutral and Si has a negative surface charge due to its low point of zero charge, pZc = 2.7).^19^

#### 2.2.2 Linker Molecule Binding

Surface positive charging is achieved by forming layers of immobilized amine-terminated linkers (3-aminopropyl)trimethoxysilane, APTMS, Sigma) and cysteamine (Cys) on Si and Au, respectively. At the experimental pH (6–7), the amines (p*K*_a_ > 9.5) should be protonated, yielding a positively charged linker layer.

For Si, the APTMS linker binds via condensation with hydroxyls on Si surface oxide.^35^ Immediately following cleaning, a Si substrate was incubated in APTMS solution with a mixture of water–methanol (MeOH) with the composition MeOH:H_2_O:APTMS = 3000:100:50 (in μL) for 5–6 h, followed by 3 min bath sonication in absolute methanol, excess Milli-Q water rinsing, and N_2_ drying, to remove any physisorbed APTMS molecules.

Cysteamine was used as a linker to the gold substrates. The substrate was incubated in 8 mg/mL (∼70 mM) of aqueous solution of cysteamine hydrochloride (Sigma) for 12 h to bind the Cys via S–Au bonds. The Cys-coated Au substrate was thoroughly washed with water followed by 1 min bath sonication with Milli-Q water to remove any physisorbed Cys molecules and N_2_ drying.

#### 2.2.3 Preparation of Protein Layers

Electrostatic immobilization of the first bR bilayer was done by incubating linker-coated substrates in 4 μM bR vesicle suspension for 12 h. Then the bR-modified substrate was rinsed with Milli-Q water and kept in the water for 12 h. Finally, the first bR bilayer was washed with water and dried with a N_2_ flow.

For bR multilayer preparation, successive bR bilayers were *covalently* bound to the first bR bilayer by a cross-linking amide bond. First, the exposed carboxylates of the immobilized bR layer were activated^19^ by 1-ethyl-3-(3-(dimethylamino)propyl)carbodiimide hydrochloride (EDC, from Sigma) treatment. A bR-coated substrate was incubated in 20 mM EDC (4 mg/mL) in 10 mM phosphate buffer with well-maintained pH (6.1)^19,36^ for 40 min under gentle shaking conditions. Then, the EDC-treated protein sample was removed. Before solvent drying, it was gently rinsed with ∼0.5 mL of Milli-Q water, avoiding excess rinsing to protect the EDC-activated carboxylic groups.

EDC-treated bR bilayer is then incubated in 4 μM of bR vesicle suspension for 12 h. In this treatment, EDC-activated carboxylate groups of the first bR bilayer are covalently bound with the amines of surface-exposed Lys residues of bR (second bilayer). As a result, two successive bR bilayers are connected via amide bonds, and importantly, the added EDC is eliminated (as a leaving group) by the process.^19^ Without EDC activation, no film growth above the first bR bilayer occurs. After this step, the sample was washed with Milli-Q water and dried with N_2_, yielding the formation of one bR bilayer on top of another. We continued the same EDC and binding process to build bR multilayers by interprotein cross-linking without using any external linker molecules in between.

Apart from the bilayer-by-bilayer control growth of the bR multilayer, we also grew, in a single step, a few thick bR multilayers (∼44 nm termed T-44 and ∼60 nm as T-60) on top of the EDC-activated first bR bilayer. The activated, first bR bilayer was then dried completely without washing, by N_2_ flow and incubated directly into the bR suspension for overnight. The resulting protein film thickness was controlled by the EDC concentration. The presence of excess EDC led to the formation of a bR multilayer by repetitive interprotein amide cross-linking. However, this approach does not provide as good layer uniformity and thickness control of the resulting bR multilayer as does the one with washing in between steps (see Figure S1).

### 2.3 Layers Characterization

#### 2.3.1 Layer Uniformity and Thickness

In ambient room temperature, ellipsometry and AFM analysis served to monitor each step of layer growth; the detailed procedure for these measurements was described in our recent work.^37^ Before forming the junction, ellipsometry-based protein layer thickness (see SI Section 1.1 in ref (37)) was used as a criterion for the suitability of the sample for junction formation.

In addition, tapping mode and scratching-based AFM imaging (using Bruker AFM setup; Nanoscope V Multimode AFM) were employed (all at ambient RT) to investigate uniformity and thickness of the bR layers (for details, see SI Section 1.2 in ref (37)). The applied contact force ranged from ∼130 to 150 nN for successful AFM scratching of the protein films,^35,37^ up to the second bR layer. With increasing protein layer thickness, successful scratching required increasing the contact force. For the triple bR bilayer the optimized contact force was ∼170 to 180 nN. For thicker layers (>40 nm) complete scratching was nearly impossible (see SI Section 1, Figure S1), even if a higher contact force of >200 nN was applied, as severe scan-related drifting occurred. Attempts to scratch away the linker layer generally failed, and therefore we report AFM thicknesses of the bR films only. The thickness of the protein layers was estimated from the depth of the line profile over the AFM-scratched region relative to the unscratched areas. Color-based height profiles of the AFM-scratched images and area-based analyses were performed to estimate the layer thickness (see SI Section 1).

#### 2.3.2 Protein Conformation

The extremely low bR concentration demands surface-enhancement IR signals for the ultrathin films on the substrate, which is possible most effectively by polarization modulation-infrared reflection–absorption spectroscopy, (PMIRRAS). However, PMIRRAS requires a reflective metallic substrate and cannot be performed for films on a Si substrate. Therefore, we used Au substrates with cysteamine as linker instead of APTMS to ensure similar protein binding to the two substrates (via the exposed amine groups). PMIRRAS measurements were done under ambient conditions with a Nicolet 6700 spectrometer (for details see SI Section 1.3 of ref (37)).

#### 2.3.3 Electrode–Protein Interfacial Electronic Structure

Ultraviolet photoelectron spectroscopy (UPS) served to investigate the frontier electronic energy levels. UPS measurements were performed with a Kratos AXIS ULTRA system, at room temperature, using the He I (21.22 eV) radiation line.^38^ The He pressure in the measuring chamber was ∼10^–7^ mbar. The total energy resolution was ∼100 meV, as determined from the Fermi edge of a Au reference sample. All UPS spectra were measured with a −10 V bias applied to the sample to enable observing the photoemission onset at low kinetic energies. The work function φ was obtained from the secondary electron cut-off (SECO) as φ = 21.22 eV – SECO, where 21.22 eV is the UV photon energy (see Figure S2, left column). *E*_HOMO_ values were deduced from the onset of photoemission (extrapolating the HOMO edge to the background signal) with respect to *E*_F_, using semilog plots (because of the low density of states in the proteins) of the UPS spectra near the Fermi level region (Figure S2 right column).^39,40^

### 2.4 Fabrication of the Protein Junctions

To construct a junction, the bR bilayers were formed on a conductive substrate. Highly doped, conductive silicon was used, which is ultraflat (rms roughness <0.2 nm by AFM) over >cm^2^ areas. A (relatively) large area, water-floated gold pad was used as the top electrode in order to enable detection of the expected low currents (ETp) through the multilayers. The large area top electrode was deposited by employing the so-called Lift-Off, Float-On (LOFO) method,^41^ using 50 nm thick, 0.5 mm diameter Au pads. Gentle blowing of N_2_ over the sample helps to achieve wrinkle-free mechanical attachment of the Au pad onto the protein layer (see Figure S3). Au LOFO pads were deposited on the substrate (Si(p^++^), serving as the counter contact), covered by a stack of regrown oxide (SiO_*x*_), linker (APTMS), and (multiple) bR bilayer(s) (Si(p^++^)/SiO_*x*_/APTMS/bR/Au). In a similar way, Au pads were placed on bare and linker-coated substrates.

### 2.5 Current–Voltage Response Acquisition

Current–voltage (*I*–*V*) measurements were performed across the different protein junctions, Si(p^++^)/SiO_*x*_/APTMS/bR/Au, via two-probe connections in a cryogenic probe station (LakeShore TTPX). Before measurements, the protein junction was kept in high vacuum (at 10^–5^–10^–6^ mbar) overnight, and this vacuum was maintained during all *I*–*V* measurements including measurements at RT. In every measurement, the bottom contact was made by having one of the probes touching a distant corner of the Si wafer that was previously scratched with a diamond cutter and fresh EGaIn (indium gallium eutectic) to ensure low-resistance (“Ohmic”) contact. Another flexible probe was landed very softly over the Au pad (top electrode).^42^ We used a sub-fA source meter (Keithley 6430) to apply the bias to the top electrode (Au) and measure the resulting current, whereas the bottom electrode (Si) was grounded.

#### 2.5.1 Room Temperature Measurements

The *I*–*V* response at 293.0 ± 2.0 K was measured using different voltage sweep scan rates; the majority of the measurements were between +0.1 and −0.1 V with a scan rate of 0.05 V/s. In addition, we also used high-voltage scans (±0.5 to ±3.5 V) at a rate of 0.5 V/s to explore possible junction breakdown. Each scan started from 0 V to the positive bias maximum, then to the largest negative bias, and back to 0 V, using dedicated LabView-based programmable software. The highest voltage (3.5 V) scan included a “breakdown” event expressed as a sudden jump of junction current from ∼200 μA to >100 mA (maximum detection limit of a Keithley 6430).

#### 2.5.2 Temperature-Dependence Measurements

The temperature dependence of the currents as a function of voltage (±0.1 V) was measured reversibly by cooling and heating the protein junction using liquid nitrogen as a coolant. For cooling, the measurements were performed sequentially from 300 to 80 K at the following intermediate temperatures: 280–240–200–160–120–100 K. Measurements were continued while the junction was gradually heated to 300 K. The temperature was controlled and measured with a LakeShore 336 temperature controller, using a silicon diode (DT-421) as temperature probe.

#### 2.5.3 Analysis by Normalized Differential Conductance (NDC)

NDC was deduced by numerical differentiation after spline smoothing for individual raw data sets. MatLab processing was done as described in SI Section 2.

### 2.6 Impedance Spectroscopy (IS)

IS measurements were done at room temperature using an impedance analyzer (Zurich Instruments MFIA). The instrument was connected to the probe station in a two-probe configuration, as described in Section 2.5. For IS we used 10 mV AC bias (amplitude) and no DC bias (0 V DC). The frequency was scanned from 1 Hz to 1 MHz, at the slowest scan rate (∼1.6 data points per second) with 20 data points per decade. During impedance measurements, data collection was done either under low vacuum (10^–3^ mbar) or under air with high humidity (>95% RH at RT) using the same chamber with a special setup (see SI, Section 3). To avoid noise at low frequency (1–200 Hz), vacuum pumping was disconnected during IS measurements, which led to a resultant moderate pressure (10^–3^ mbar). Except for the measurement under a high-humidity environment, all IS data were collected in low vacuum for all bR bilayers. Impedance (*Z*) data were further analyzed by fitting the plot of the negative of the imaginary *Z* component (−*Z*_im_) vs the real component (*Z*_re_), a so-called Nyquist plot, to an equivalent circuit using Z-view 3.2b software (Scribbner Associates) with good fitting agreement (χ^2^ < 0.004), as discussed below in Section 3.9.

## 3 Results and Discussion

### 3.1 Protein Layer Characterization

**Figure 2:**
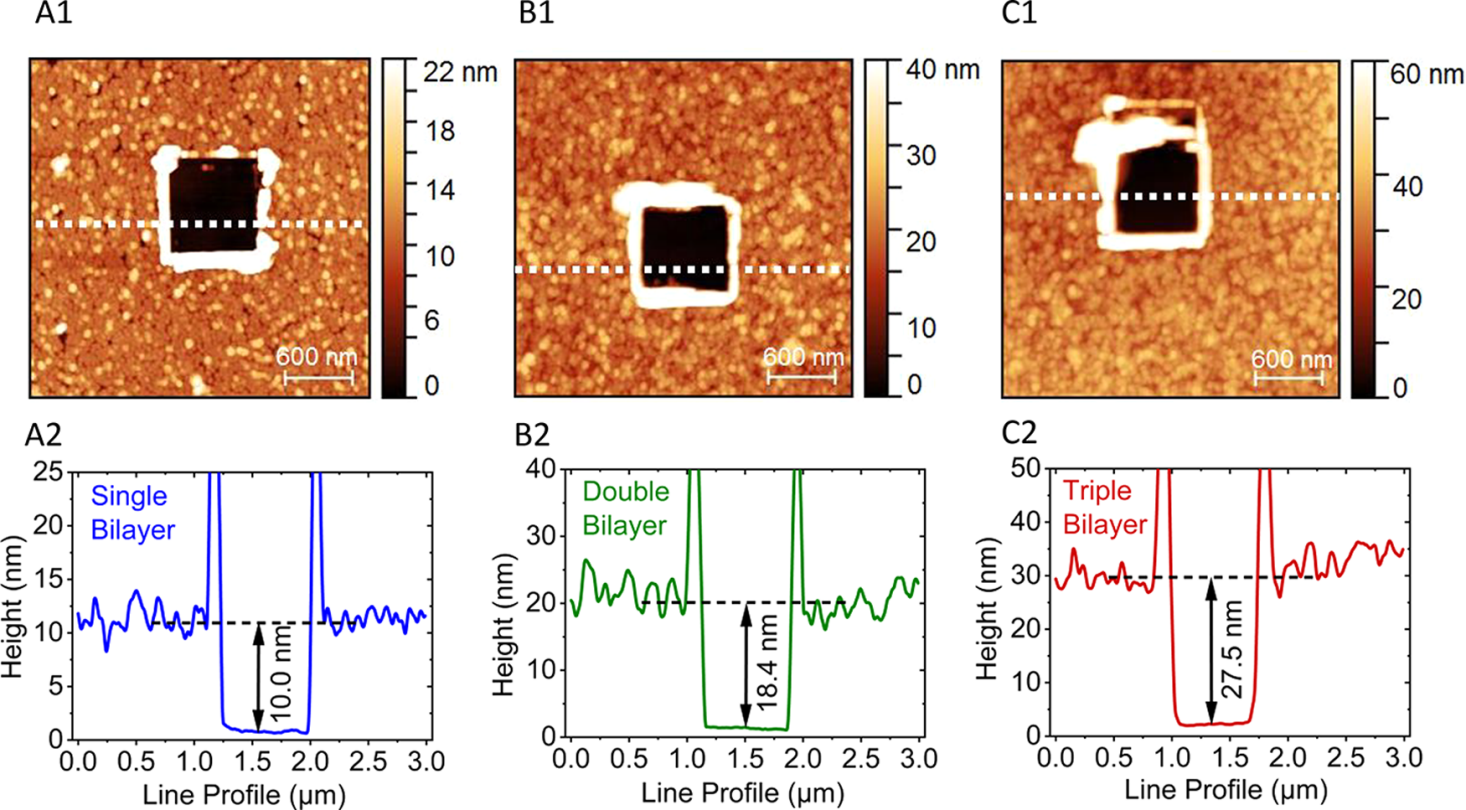
AFM scratching images and cross sections from line profile scans of different bR bilayers on p^++^-Si/SiO_*x*_ substrates, showing topography (top row) and line profile (bottom row): A1, A2 for single bilayer; B1, B2 for double bilayer; and C1, C2 for triple bilayer. The black squares in the top row are the scratched regions. The white dotted lines indicate where the line profiles (shown in the bottom row) were measured. The profiles show the depths of the scratched regions for each of the samples in the top row. Scan areas are 3 × 3 μm^2^ in all cases, and the color scales vary from 0 to 22 nm, to 40 nm, or to 60 nm for the A1, B1, or C1 panels, respectively.

The composition and growth of bR layers, as described in the Experimental Section, are schematically presented in Figure 1. The thickness of the bR layers was estimated from ellipsometry and AFM scratching experiments (Figure 2 and Table 1). The measured thickness range was 0.8–1.0 nm^28^ for the regrown silicon oxide, 0.4–0.6 nm for linkers (APTMS or Cys), and 8.0–10.0, 15.0–19.0, and 23.0–29.0 nm for the single, double, and triple bR bilayers, respectively, by ellipsometry. The thicknesses of the protein films were found to increase linearly with the number of successive bR bilayers (Table 1). The bR multilayer thickness was close to an integral multiple of the width of a single bilayer. Thus, the successively formed bR bilayers can be designated as single, double, and triple bilayers. We also prepared bR multilayers with thickness of ∼44.0 nm (T-44) and ∼60.0 nm (T-60) (details in Section 2.2.3).

**Table 1:** Thickness (in nm) of the Single, Double, and Triple Bilayer bR Films, Derived Independently from Ellipsometry and AFM

	thickness (nm)	
protein layer	ellipsometry^a^	AFM^b^
single bilayer	8–10	9–10
double bilayer	15–19	16–20
triple bilayer	23–29	25–31

a Individual thickness of linker and SiO_*x*_ was fitted and entered as a fixed separate thickness parameter (from protein layer) in the ellipsometry model.

b Linker + SiO_*x*_ resist tip scratching and therefore do not add to the trench height (see Section 2.3.1).

The linear increase in thickness with the number of bR bilayers suggests a reasonably regular bR bilayer building on top of each other on the substrate (Table 1). This fits with (similar) dense uniform protein coverage over both Si and Au substrates that was observed by AFM topography measurements (Figures 2, S4, S5, S6, and S7).

The thickness values, deduced from ellipsometry (over an area of ∼10^–1^ cm^2^), were also measured by AFM (on an ∼10^–9^ cm^2^ area), and the results are closely comparable, indicating uniform layer thickness (protein coverage) all over the substrate. Different analyses of the AFM-scratching results (Figures 2, S1, S4, and S7), yielded comparable films widths. The average thickness of the repeating bR unit bilayer was ∼9.0 nm, i.e., close to twice the long dimension of bR, derived from its crystallographic (PDB 1FBB or 1BRR) structure (∼5.0 nm).^21^ Therefore, the repeating bR unit can be considered equivalent to a single bR bilayer.^26^

We lack direct evidence for how the two bR monolayers are connected, given that the bilayer originates from the collapse of the bR vesicle. The vesicle collapse is likely caused by shrinkage of the surface-immobilized (doughnut-like^24^) bR vesicles upon drying on the substrate surface. The experimentally derived width of the first, thinnest bR layer is much too large to be consistent with monolayer formation, which would result from vesicle fusion. The inner, monolayer–monolayer, interface of the bR bilayer is formed by the cytoplasmic surfaces of the bR membranes; i.e., the protein trimers (PDB 1BRR) are surrounded by two back-to-back native lipid bilayers (lipid tetralayer), as shown schematically in Figure 1C. We posit that the interaction at the inner interface of the bilayer is by both van der Waals and electrostatic interactions between the polar head groups of the surface-exposed parts of the protein–lipids complex and the counter charges. Electroneutrality dictates that those charges (counterions) accompany the protein during film deposition on substrates from solution. We assume that no trapped water remains between the collapsed vesicles under the high-vacuum conditions of our electrical transport measurements.

**Figure 3:**
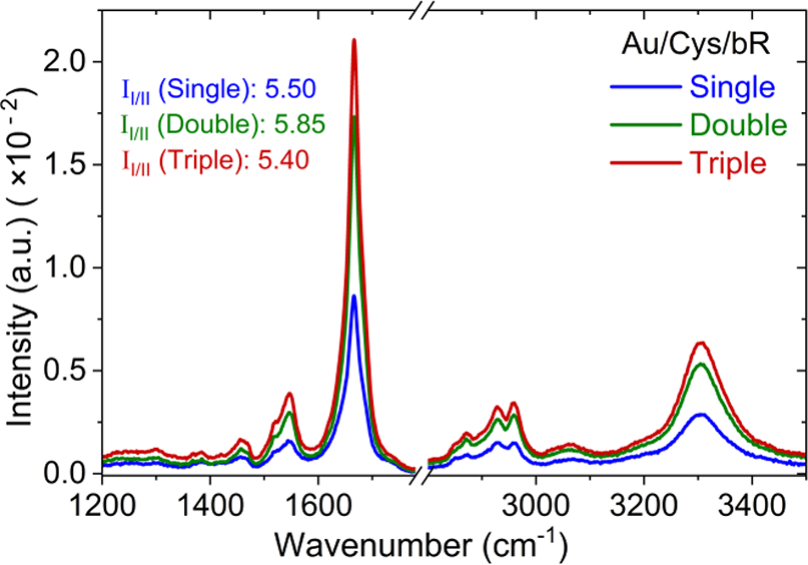
PMIRRAS spectra for different bR bilayers (single, double, and triple) on cysteamine-coated gold substrates (Au/Cys/bR), showing identical peak positions. Those of the amide I and amide II peaks are at 1667 and 1547 cm^–1^, respectively. The intensity ratio of amide I/amide II peaks (*I*_I**/**II_) is ∼5.5, irrespective of the thickness of the protein layers (see left legend in the figure).

The nature and integrity of bR in the adsorbed films were characterized by PMIRRAS measurements for different bR bilayer thickness (single, double, and triple bilayers) as shown in Figure 3. Increasing the amount of bR on the surface only increased the intensity of obtained IR spectra, while all show bands at identical wavenumbers, including the informative amide I (∼1667.0 cm^–1^) and amide II (∼1547.0 cm^–1^) bands. Furthermore, the amide I to amide II peak intensity ratio was also similar (∼5.5) and independent of the number of bR bilayers (legend to Figure 3). This is a clear indication that the protein conformation is preserved among the multiple bilayers of bR.

### 3.2 Electron Transport at Room Temperature as a Function of bR Film Thickness

**Figure 4:**
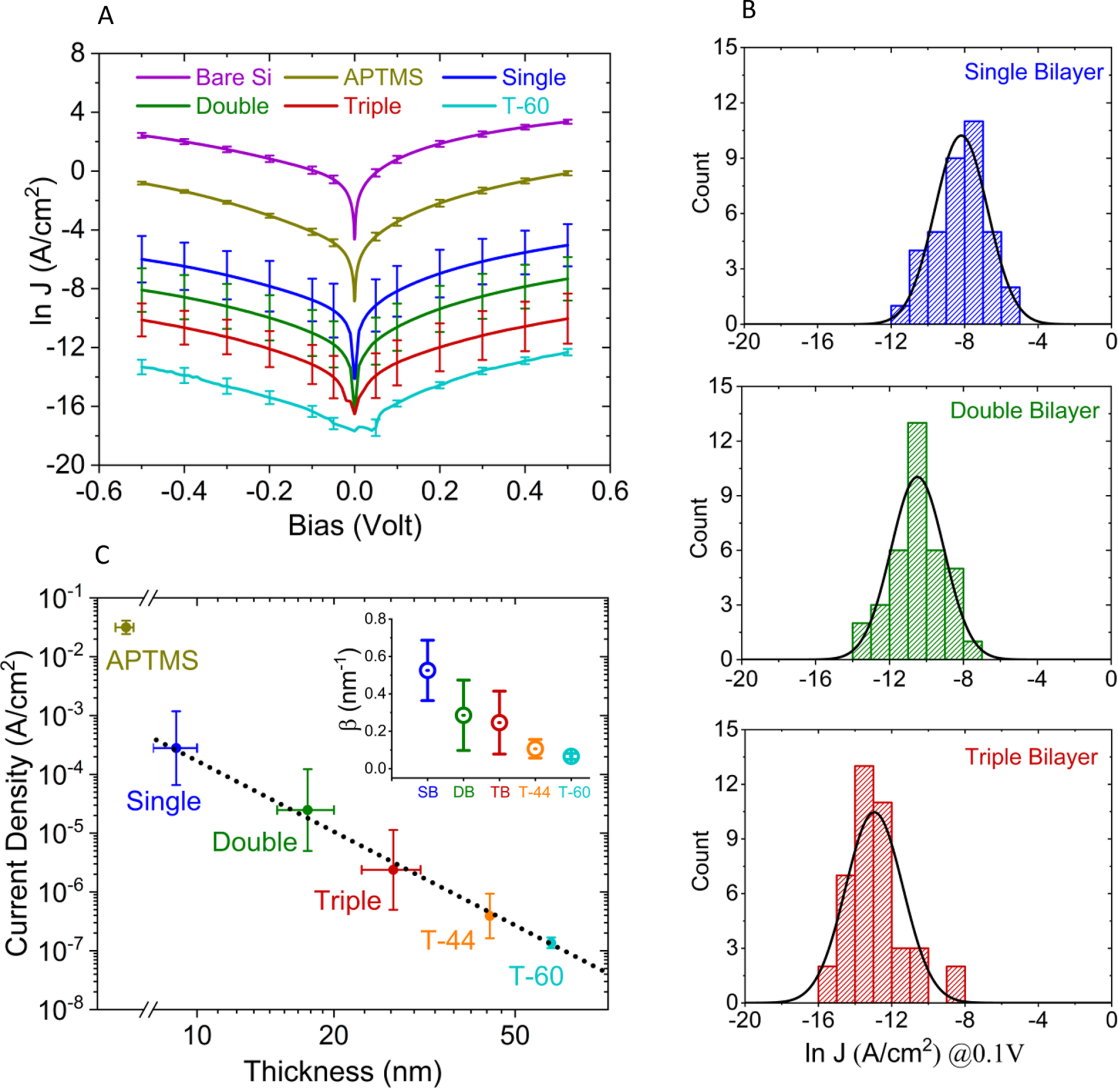
Observed currents across the p^++^-Si/SiO_*x*_/APTMS/bR/Au junctions with increasing bR multibilayer film thickness. (A) Mean ln *J* vs *V* plots for junctions with only p^++^-Si/SiO_*x*_ (regrown SiO_*x*_ is <1 nm thick), SiO_*x*_ coated with APTMS, and those further covered with the three types of bR bilayers and with the 60 nm thick (T-60) bR multilayer, as indicated in the legends. All measurements were performed in a vacuum (10^–5^ mbar; 10^3^ Pa) at RT (293 ± 2 K). (B) Distributions of junction current densities, *J*, at +0.1 V bias, for single, double, and triple bilayers; *J* is derived from the measured currents by using the geometric surface area of the Au pad contact. The distributions of the raw ln *J* data (histograms in panel (B)) are fitted to Gaussian distributions (solid black lines). The same process was repeated for all measured data, and the mean (center) and the standard deviation are used in (A) and (C) for values and errors, respectively. (C) A log–log plot of current density (*J*) vs bR layer thickness, yielding a straight line with a slope close to −4 (the black dotted line is a linear fit with *R*^2^ = 0.99), as further discussed in the text, for the junctions presented in (A), and of an additional junction with a 44 nm thick bR (T-44) multilayer, measured at 0.1 V. The error in the thickness (in panel (C)) is defined by the net range of thickness variation (obtained from ellipsometry). Inset to panel (C): values of the standard, monoexponential distance decay constant (*β* in nm^–1^), derived from the slopes of ln *J* vs thickness (*r*) (using eq 1 and SI Figure S8). *β* serves here as an empirical parameter.

Room temperature current–voltage responses were measured via the different bR junctions to extract the dependence of the measured currents on the junction length (distance between Au and Si (p^++^) contacts; Figure 1B). Junction current statistics (in terms of current density, *J*) were made for each type of bR bilayer using ∼40 different protein junctions at 100 mV applied bias (Figure 4B). Clear differences in the most probable junction currents (peaks of the distributions), from 10 to 10^4^, were observed between the different types of bR bilayers. The average conductance of a single bilayer is ∼2 orders of magnitude less than that of the linker (APTMS)-only junction (Figure 4A). The T-60 bR (i.e., ∼60.0 nm) junction showed 4 orders of magnitude (at 0.5 V) lower current than the ∼9.0 nm, single bR bilayer junction. Still, the tails of the current histograms (Figure 4B) or error bars (in Figure 4A) overlap with the opposite tails of bR junctions that are thicker or thinner by one bilayer. The left tail of the histogram can be consistent with that of junctions that are thicker than the average, which can be ascribed to protein aggregation. The right histogram tails are likely due to junctions that are in part thinner because of voids in the top layer (the issue of possible pinholes is discussed in Section 3.5).

The current density (*J*) as a function of interelectrode distance (*r*), taken here as the thickness of the bR layer, is shown in Figure 4C. The dependence of the current decrease with increasing distance follows a *J* ∝ 1/*r*^4^ relation (see log–log scale of Figure 4C), rather than the exponential decay, expected for tunneling (eq 1). For comparison reasons, we have extracted several *β* values (see the inset of Figure 4C) for each of different thickness (*r*) of bR bilayers from the differential slope (d ln *J*/d*r*) in Figure S8.

The approximated *β* values (from eq 1) are 0.5, 0.3, 0.25, 0.1, and 0.05 nm^–1^ for a single, double, triple bilayer, T-44, and T-60 multilayer junctions, respectively (inset to Figure 4C). These *β* values, especially of the thicker multilayers, are low compared to those obtained for other, well-studied proteins that are efficient ETp media, particularly PS-I (0.8–1.6 nm^–1^),^43^ models of STC protein stacks (2 nm^–1^),^44^ azurin (1.8 nm^–1^),^28^ and ferritin (0.3–1.3 nm^–1^).^45^ To make a more consistent comparison, *β* values were extracted from results obtained with the same protein junction configuration (Si/SiO_*x*_/linker/protein/Au-pad), all at 0.1 V applied bias, and plotted against the experimentally derived different protein films’ thickness values (Figure S9). The obtained low *β* values (for bilayer junctions) seem to be the characteristic of bR junctions, implying that it is a highly efficient long-range electron transport medium among the different protein biomolecular junctions studied so far. The question arises as to why the *β* values for bR are an order of magnitude smaller than values found for other proteins. Compared to the latter, the α-helix-rich nature of bR might boost ETp efficiency, as previously shown for peptides.^46^

The surprising 1/*r*^4^ length dependence is predicted by quantum space charge-limited current (Q-SCLC), guided by the quantum Child–Langmuir (CL) law relation.^47,48^ The space charge potential barrier near the electrode could lead to electron tunneling through the high dielectric bR medium.^48^ However, Q-SCLC predicts sublinear voltage dependence (), in contrast to what we find experimentally, which varies from linear (*V*^1^) at low voltage to mildly polynomial as the voltage increases (see Section 3.7). Furthermore, the intriguing 1/*r*^4^ dependence cannot be reconciled with either multiple sites or rate equations; it may, though, indicate a key role of charging effects in bR junctions and possibly other biomolecular junctions, which requires further studies.

### 3.3 Temperature Dependence of the ETp

**Figure 5:**
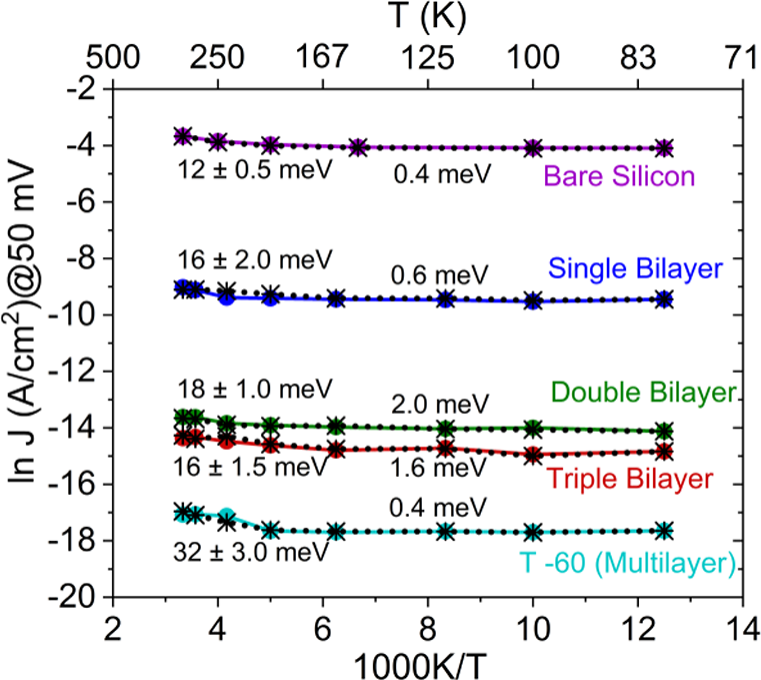
Plots of ln *J* vs 1/*T* over the 80–300 K temperature range at +50 mV applied bias for p^++^-Si/SiO_*x*_/APTMS/bR/Au junctions with different thickness of protein bilayer (different color for each), including the 60 nm thick bR multilayers (T-60), and of the bare p^++^-Si substrate, covered with <1 nm oxide. The results are both for cooling (solid colored line) to 80 K and for heating back up (black dotted line) to 300 K, which closely overlap. The big color dots and black stars represent different experimental temperatures between 80 and 300 K. Values next to each line state the trend slopes, corresponding to thermal activation energies, divided into near RT (left side) and one or more orders of magnitude smaller at lower temperatures (<160 K, right side). All data were collected in a 10^–5^–10^–6^ mbar vacuum in the probe station.

To investigate the mechanism of ETp, *I*–*V* scans were acquired for the different bR junctions over the 80–300 K temperature range by reversible cooling and heating (see Section 2.5.2). More than 35 protein junctions with different protein thicknesses were used in these studies: 14 for single bilayer, 9 for double, 10 for triple, and 3 for the T-60 multibilayers (for the T-44 temperature dependence was not studied). The variation of current at 0.05 V as a function of reciprocal temperature (Arrhenius) is shown in Figure 5. For all junction lengths, the observed currents below ∼160 K, were, at a given voltage, essentially temperature independent (i.e., apparent activation energies, *E*_a_, ≤2.0 meV, which is even smaller than the lowest thermal energy, *k*_B_*T* at 80 K ∼ 7.0 meV). Above 160 K, a small positive *T*-dependence was observed (Figure 5), which, except for the T-60 junction, differs insignificantly from *E*_a_, found for Si/SiO_*x*_/Au junctions, i.e., without a protein effect. In this temperature range *E*_a_ (derived from the Arrhenius plots) ranged from ∼16.0–32.0 meV (∼0.6–1.3 × *k*_B_*T* at RT) (Figure 5). Thus, considering *I*–*V*–*T* data obtained via the bare substrate as a baseline, the actual *E*_a_ via the protein is even smaller, becoming at most, i.e., near RT, 0.7 × *k*_B_*T* (for T-60). Furthermore, the estimated *E*_a_ for current across our bR bilayer junctions varies insignificantly within our experimental voltage range (±0.1 V) (see Table S1). This result begs the question how transport can occur without significant thermal activation over such distance, comparable to 100s of C–C bonds, and, essentially, excluding hopping as possible ETp mechanism.^12^ In addition, most hopping barriers observed for organic molecular junctions are well over a few 100 meV.^49^ We note that also pure tunneling has a slight temperature dependence because occupation of the energy levels above *E*_F_ increases with temperature, as has been experimentally verified for Si/SiO_*x*_/Au junctions of different SiO_*x*_ widths.^50^ However, the Si/SiO_*x*_ junction baseline temperature dependence at higher temperatures, and its similarity to the *T*-dependence of the protein junctions in the same temperature range, yields a net protein junction behavior that differs from the temperature dependence, expected for direct tunneling.^50^

### 3.4 Protein Energy Levels Relative to the Electrode Fermi Level

**Figure 6:**
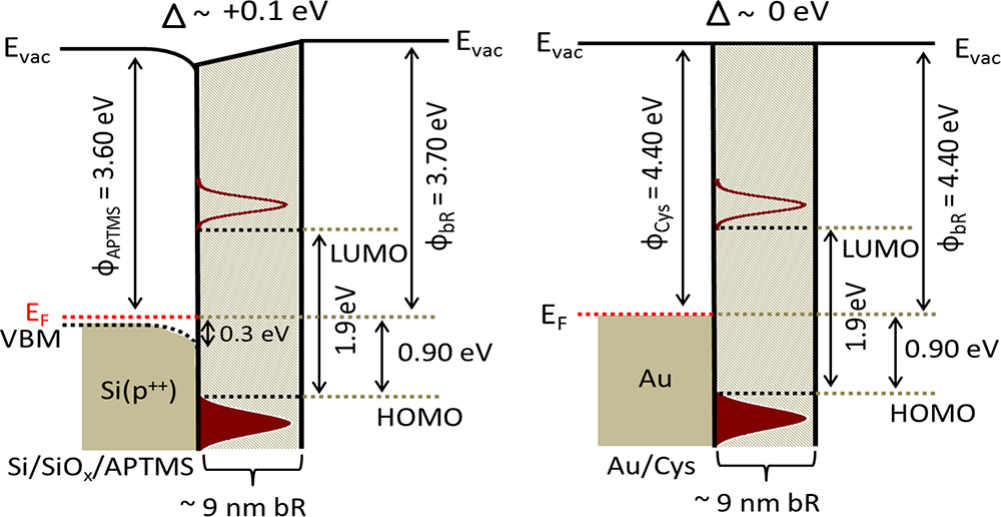
Energy diagram of ∼9 nm bR single bilayers adsorbed on top of p^++^-Si/SiO_*x*_/APTMS (left) or Au/cysteamine (right). Here we do not consider the separate energy levels of the thin SiO_*x*_ and linker layers as their combined width (<2 nm) is well within the tunneling regime for the currents that pass. φ is the relevant work function, including linker (as suffix) as measured by UPS; *E*_vac_ is the vacuum level; and Δ is the change in *E*_vac_ after protein deposition on the respective linker-coated electrodes. The red dotted line represents the system Fermi level (*E*_F_). VBM is the valence band maximum of heavily doped p-type Si, just below *E*_F_ (∼0.05 eV by nominal resistivity). UPS indicates that upon APTMS adsorption, the silicon VBM shifts downward by 0.3 eV relative to *E*_F_ (see SI Figure S2). Dark red Gaussians represent substrate-broadened molecular HOMO (filled) and LUMO (empty), with their edges (black dotted lines) being the physically critical values. The position of the HOMO energy level is obtained from the UPS data; the LUMO energy level is deduced from the HOMO level, and the forbidden energy gap is deduced from the optical absorption spectrum. Notice that these energy diagrams refer to “half-junctions” without a top electrode.

The electronic energy barrier at the protein–electrode interface that electrons must cross plays a crucial role in ETp through protein junctions. Figure 6 shows the energy diagrams of the two types of bR modified surfaces (or bR/substrate films) used here. The approximate energies of the electrode Fermi level and protein HOMO level were extracted from the secondary electron cut-off (SECO) (Figure S2, left column) and from the low (binding) energy onset of the UPS spectra (Figure S2, right column), respectively. While the LUMO energy level can in principle be obtained from inverse photoemission spectroscopy (IPES), this method is unsuitable for proteins because of their low density of states, the limited sensitivity of IPES, and the electron beam sensitivity of proteins. Instead, the LUMO level was estimated by combining the UPS-measured HOMO level and UV–vis absorption spectra for the HOMO–LUMO gap. Previously, Ron et al.^28^ showed that the UV–vis absorption of bR was nearly identical in solution and in the employed ultrathin film configuration.^28^ The HOMO–LUMO gap (∼1.9 eV) was estimated from the longer wavelength absorption edge of bR (see SI Section 4, Figure S10). Here the designated LUMO in Figure 6 corresponds to the position of the lowest unoccupied level of the retinal, embedded in the bR (see Figure S10).

All bR junctions were made between two different electrodes as contacts, viz. conductive silicon and gold electrodes. UPS measurement is not realistic for the whole protein junction because the escape depth of electrons is, at best, a few nanometers, namely at least an order of magnitude less than the top contact thickness. To understand the influence of the electrode on bR layer, a single bilayer was prepared on both electrodes via amine-terminated linkers, as described in the Experimental Section. The UPS data yield the protein’s HOMO level relative to the electrode Fermi level. Because of severe charging effects, we could obtain reliable UPS results only for single bR bilayer films. *E*_F_ of cysteamine-coated Au is almost midway between the HOMO and LUMO energies of bR (∼1 eV). The situation is similar on (linker-coated) p^++^-Si (Figure 6). Interestingly, in complete junctions, currents could be measured even at close to 0 V despite the large injection barrier (∼1 eV) that we deduce from the UPS data. Thus, in this low-bias applied scenario, no energy levels of the protein frontier orbitals are equi-energetic with the *E*_F_ of the electrode, unless they form during top-contact formation (note that no chemical bonds are formed; top contacts are deposited mechanically without linker molecules on them). Therefore, at low bias, nonresonant tunneling^51^ could, in principle, be a mechanism for transport *across the* junction, if it was not for the large separation between contacts. Thus, at low applied bias (<100 mV), the absence of energy levels between the protein HOMO (or LUMO) and the electrode (quasi)-Fermi level rules out hopping as the ETp mechanism via the bR junctions for the temperature range under study.

### 3.5 Effective Transport Length across the bR Junctions

**Figure 7:**
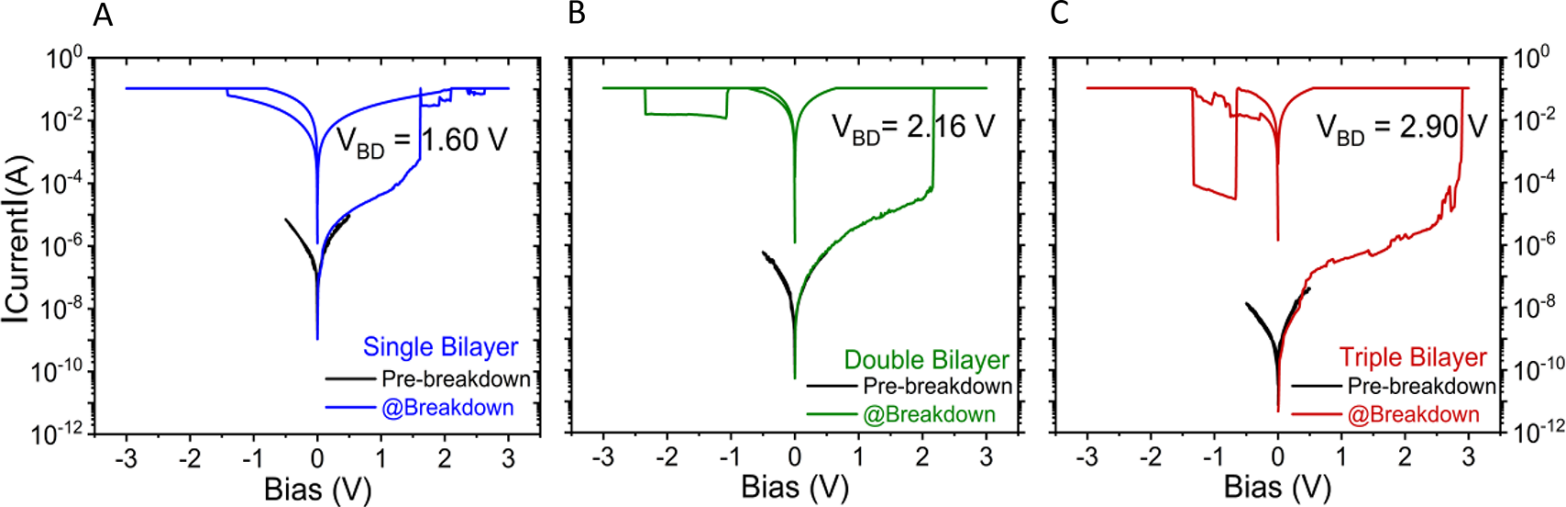
High-voltage breakdown of p^++^-Si/SiO_*x*_/APTMS/bR/Au junctions for (A) single, (B) double, and (C) triple bR bilayers, showing an initial ±0.5 V scan (black) followed by ±3.0 V scan (colored). Each plot shows a sharp current rise (almost vertically) at a particular bias, designated as the breakdown voltage (*V*_BD_). *I*–*V* traces are representative; the scan sequence is 0 → positive → negative → 0. We can speculate that for the thicker bR bilayers, the sudden drops and increases of junction current in (B) and (C), at negative applied bias, might correspond to filament rupture and regrowth across the bR bilayers. The data were collected under vacuum (10^–5^ mbar) at RT (293 ± 2 K) in a probe station.

Pinholes can, in principle, play a role in leakage currents, but interstitial voids of several nanometers are unlikely for monolayers of small molecules like linear alkylthiols. At the same time, this cannot be excluded, even for densely packed layers of proteins, including bR, and may lead to lower breakdown voltage (*V*_BD_). AFM and ellipsometry-based measurements on the protein films before top-contact deposition show a linear increase in thickness on the substrate with the number of added bilayers. However, in view of the conclusions that neither tunneling nor hopping can explain the results, the question arises of whether the protein layer separation is maintained after top electrode deposition. We investigated this issue by measuring high-field-induced breakdown of junctions that depends on the dielectric constant of the medium and the separation length between the electrodes. Breakdown tests were performed on protein junctions of varying thickness, as shown in Figure 7. The breakdown voltage is a qualitative indicator of the effective electron transport length because the likely slight decrease in dielectric constant as the bilayer quality decreases (more voids) will be negligible compared to the changes in electrode separation.

The specific bias value at which the junction current was observed to suddenly increase was considered the breakdown voltage (*V*_BD_) of the junction. The *V*_BD_ of the single-bilayer junctions was 1.4–1.6 V, 2.1–2.3 V for the double bilayer, and 2.8–3.0 V for the triple bilayer (Figure 7). A plot of the characteristic breakdown voltage against junction thickness (*r*) is linear (see Figure S11), and the electric field value is derived from the slope of the plot as ∼0.1 GV/m. As this breakdown field (*V*_BD_/*r*) is constant (within experimental resolution) it implies that the *effective* transport distance does *not* decrease with increasing width of the protein bilayer, strong evidence against ETp via pinholes. These *V*_BD_ values correspond to electric field strengths that are about an order of magnitude less than that reported for the thinner alkanethiol system.^52^ The higher effective dielectric constant of a protein^53^ than of alkylthiols rationalizes the higher *V*_BD_ for the latter.

Earlier we showed^54^ (in the SI, Figure S9 of ref (54)) that the possibility of leakage currents through pinholes will be negligible for LOFO contacts on layers of the closely related protein halorhodopsin, then thought to be monolayers. For a bilayer, the probability of significant current contribution through the pinhole contacts only decreases.

Therefore, the estimated *V*_BD_ values for different bR bilayers qualitatively confirm the increase of the protein films’ width, i.e., the path that electrons need to transit, increases as more bR bilayers are added to the junctions, rather than, e.g., the pinhole influence on junction conductivity.

### 3.6 Control Experiment: Comparison with PMMA Junction

To examine whether one can simulate the possibility of measuring artifacts, we replaced the triple bR bilayer (and the T-60 bR junction) with the thinnest layer of a saturated polymer that we could reproducibly make, namely, an ∼30.0 nm spin-coated poly(methyl methacrylate) (PMMA). We could not detect current above the 0.5 pA background noise through the (p^++^-Si/SiO_*x*_/PMMA/Pb–Au) junction, even in a wide voltage sweep (±1.0 V) (data not shown). We used an evaporated Pb–Au top electrode because depositing an Au pad on a PMMA layer was problematic, as it always led to broken/cracked Au pads full of wrinkles.

The lack of detectable current cannot be attributed to the use of Pb–Au rather than Au-LOFO for the top electrode. We have earlier compared these two alternative top contacts to bR^55^ (Figure 4A in ref (55)) and found that ETp via bR junctions with evaporated Pb–Au top electrode yielded conductance values that are similar to or even higher (at low voltages) than those obtained with LOFO Au pads. We also repeated the measurement on different bR bilayer (p^++^-Si/SiO_*x*_/APTMS/bR/Pb–Au) junctions (Figure S12) with a similar trend of results to that for the earlier report^55^ on a single bilayer. Thus, the blocking of current through a 30.0 nm PMMA film in a junction configuration as that used for the bR films adds to the evidence for actual electronic conduction through the bR junctions. It also excludes significant conduction via any remaining lipids (completely saturated organic matter, as is PMMA).

The current obtained with the T-60 junction (∼500 pA at 0.5 V) was still >3 orders of magnitude higher than the system’s current noise level. Therefore, a thick (∼60 nm) bR layer (T-60) is more than 3 orders of magnitude more efficient as an electronic transport medium than an ∼30 nm PMMA layer.

### 3.7 Normalized Differential Conductance of the bR Junctions

**Figure 8:**
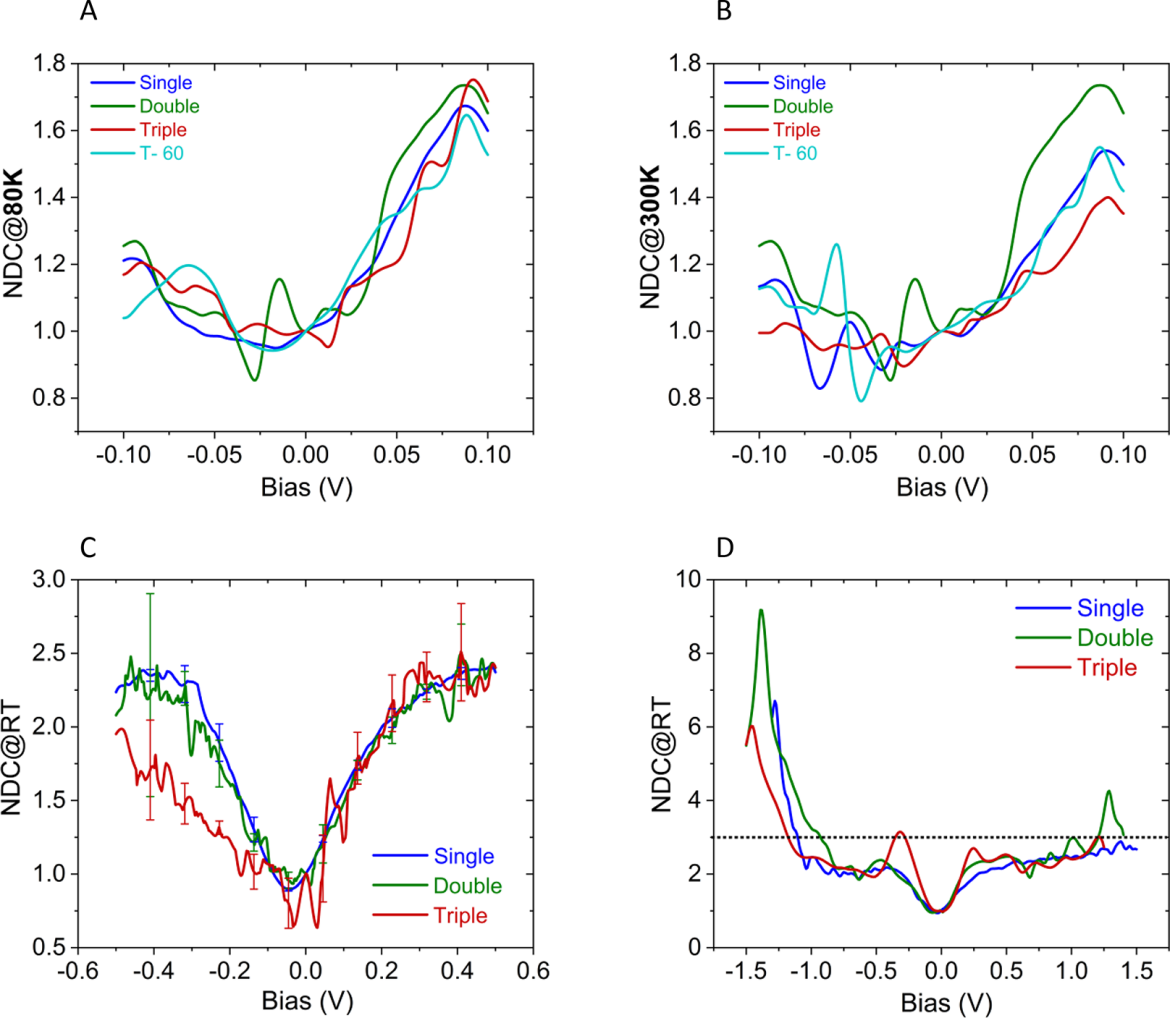
Normalized differential conductance (NDC) plots (see main text), derived from the experimental *I*–*V* plots, shown in Figure S13, between −0.1 V and +0.1 V at (A) (80 K) and (B) (300 K) for the different bR bilayers (single, double, and triple, and T-60). The room temperature NDC characteristics for two additional, higher applied bias voltages are shown in (C) at ±0.5 V and (D) at ±1.5 V for three types of bR junctions. In (D) the dotted line indicates NDC = 3. Data were collected under vacuum (10^–5^–10^–6^ mbar) at RT.

Transport across (bio)molecular junctions is generally characterized by a weak-voltage dependence, which varies between linear dependence (“Ohmic”) to low-polynomial dependence (*I* ∝ *V*^*p*^, with *p* ∼ 3). In contrast, the linear part of the *I*–*V* characteristic, between approximately −50 and +50 mV, varies by orders of magnitude because of exponential length decay (eq 1 and Figure S8). Normalized differential conductance (NDC) is a simple tool to eliminate this orders-of-magnitude first-order (linear) contribution and magnify the higher-order voltage effect, providing quantitative insight into the transport mechanism. NDC is the ratio between a junction’s differential conductance (d*I*/d*V*) and the overall conductance (*I*/*V*). For an ideal Ohmic contact (linear *I*–*V*), NDC = 1 irrespective of applied bias. However, any deviation from linearity of the *I*–*V* shows up clearly as NDC ≠ 1 with some voltage dependence.^56^ The NDC analyses of bR junctions derived from their respective *I*–*V* curves (Figure S13) are shown in Figure 8. The voltage-dependence of NDC is shown at two representative temperatures (80 and 300 K, Figures 8A and 8B) over a low-voltage scan and at room temperature for increasing voltage ranges (Figures 8C and 8D) for different bR junctions (curves in each panel).

Further to our discussions on hopping in Sections 3.3 and 3.4, we also used the NDC analysis in order to examine the possibility that ETp is dominated by hopping, which is expected to increase exponentially with the applied voltage^57−59^
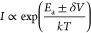
2yielding a linear NDC–*V* dependence^56^
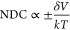
3where ±δ*V* expresses the fraction of the applied voltage that drops over the hopping site.^59^ Notably, the voltage effect on hopping is expected to scale with temperature, and therefore if the transport mechanism was hopping-dominated, one would expect to observe a stronger voltage dependence of NDC at low *T* (Figure 8A) than at higher *T* (Figure 8B). In addition, experimental NDC saturates for |*V*| > ∼0.3 V (Figures 8C and 8D), except for extreme negative voltage. NDC saturation is typical for a polynomial dependence (with a higher *p* ≈ saturation, ∼2.5 here). Therefore, the obtained voltage-dependent NDC behavior is *inconsistent* with hopping-dominated ETp.

In addition, NDC analyses show that the higher-order voltage effect is *independent of the thickness* (number of bilayers), as shown by the overlap of the NDC–*V* traces in Figures 8A and 8B. This overlap is seen, notwithstanding the 3 order of magnitude difference in the net current at 0.1 V via the single bilayer and T-60 junctions (Figure 4C) and the ∼7-fold smaller electric field across the thickest bR multilayer than that over the single bilayer. The absence of a measurable higher order voltage effect of junction length on the NDC excludes (its fit to) WKB-type (tunneling) models.^60,61^ Therefore, the currents seem to be voltage-dependent rather than field-dependent, which fits with a rate-limiting step that is independent of the film thickness and is relatively localized.

The localization can be understood if the voltage drop is across one or both contacts of the electrodes. Upon increasing the applied bias (within ±0.5 V), the NDC of the bR junctions increases gradually and reaches a plateau above ∼0.3 V with NDC value being ∼2.5–3 (Figures 8C and 8D), a behavior that is often observed for junctions that are thought to be dominated by tunneling.^60^ Below ∼−1 V, the NDC exceeds 3 (Figure 8D) with a strong voltage dependence, indicating a clear change in transport mechanism, likely to “hopping-dominated” ETp. Here the retinal energy band (see SI Section 4, Figure S10) could play a crucial role (considered as LUMO in Figure 6) in the ETp at applied high bias conditions. In the protein layer matrix, the retinals are separated by 2–3 nm (horizontally, i.e., in parallel to the electrode surfaces). As we apply bias to the top electrode (Au) for the measurements, therefore, at high applied bias, the retinal’s energy levels can take part as intermediate electronic states between the electrodes. At ∼−1.0 V, the Au Fermi level is within the retinoid band (“LUMO” of Figure 6), which could assist electron hopping through the discontinuous protein energy levels.

### 3.8 Which Contact Dominates the p^++^-Si/SiO_*x*_/bR/Top-Electrode Junction?

There is good agreement between the NDC onset and UPS energy difference, viz., about 1.0 eV between *E*_F_ and either HOMO or LUMO, according to the scheme in Figure 6. This agreement suggests, apart from the fact that deposition of the top contact does not significantly alter the level alignment, an asymmetric voltage drop over a single interface, not over both. From the earlier literature, the actual material of the top electrode contact on junctions of protein films, immobilized on Si, does not influence the overall junction conductivity. Thus, Hg (drop) and Au (LOFO) as top electrode on Si-bound proteins showed similar ETp.^55^ In the present work, we replaced our Au (LOFO) top electrode with evaporated Pb, followed by a thin (oxidation-protecting) evaporated Au layer for single, double, and triple bilayer junctions. While on single bilayers the still aggressive thermal evaporation process caused most junctions being shorted, for the thicker films sufficient junction histograms were obtained (Figure S12A). A representative *I*–*V* plot of a few single bilayer junctions that survived in Pb evaporation is shown in Figure S12B. The results are comparable to those obtained with Au (LOFO) contacted junctions.

Based on these results, we conclude that the silicon contact to the protein plays a dominant role in ETp through these bR junctions. Then, a negative voltage applied to the Au (Si is grounded) can bring Si into resonance with the HOMO of bR and give HOMO-dominant transport. Moreover, the onset (∼−1.0 V in Figure 8D) observed only at negative *V* and the lack of it at positive *V* indicates that one channel contributes while the other does not, at least within the experimentally tested voltage range.

### 3.9 Impedance Spectra of bR Junctions

**Figure 9:**
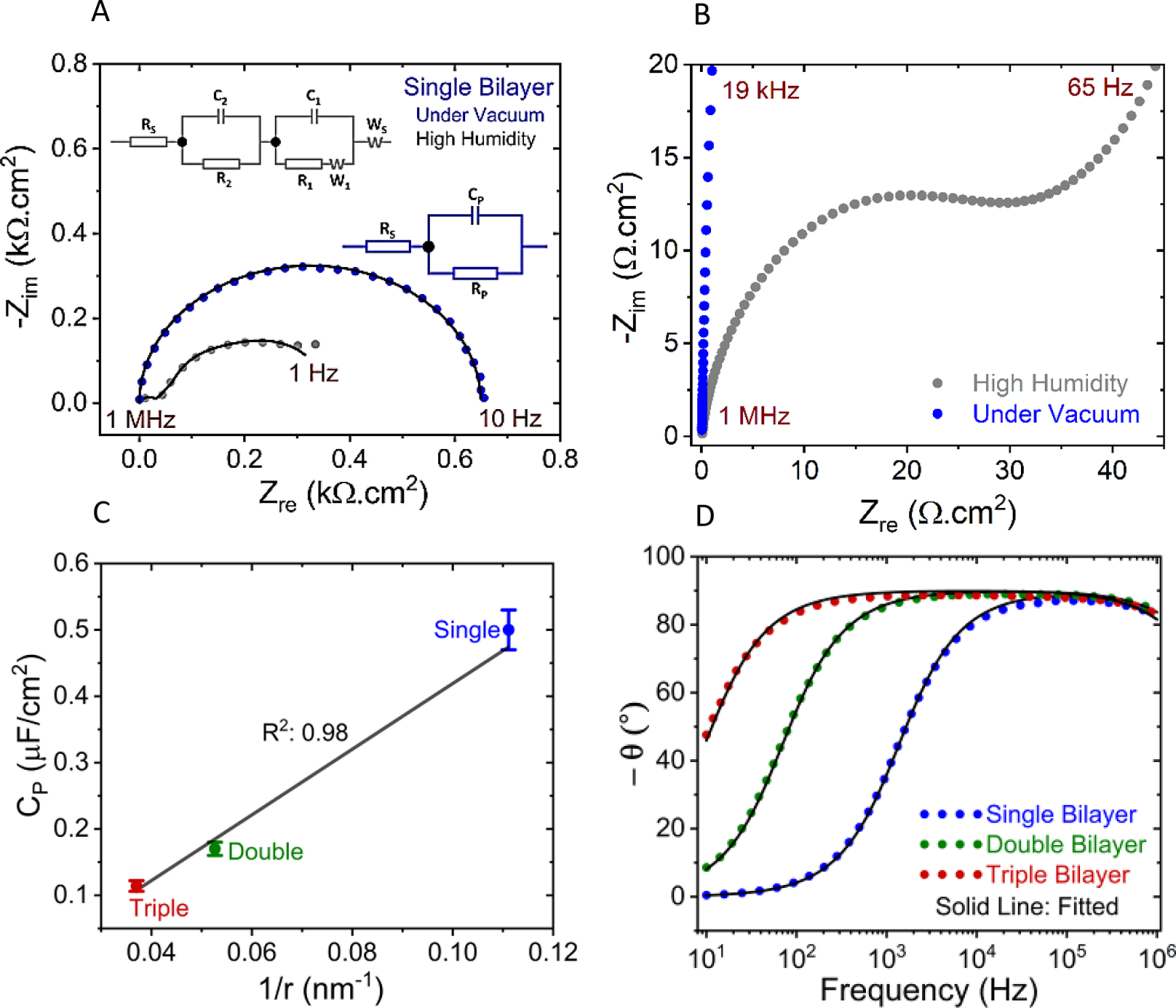
(A) Impedance-based Nyquist plot (negative of imaginary part (−*Z*_im_) vs real part (*Z*_re_) of impedance) for p^++^-Si/SiO_*x*_/APTMS/bR/Au junctions with a single bR bilayer, in high humidity air at atmospheric pressure (>95%, gray dots), and in a vacuum (10^–3^ mbar, blue dots), both at room temperature (293 ± 2 K). The solid lines are the fits to equivalent circuits, shown as insets in the plot in the corresponding colors, blue at the right for the measurement in a vacuum, and gray at the top-left for that under high humidity. Equivalent circuit elements are described in Section 3.9, and the values of the parameters for the fits are given in Tables S2 and S3 in Figure S14. (B) ZOOM-IN of Nyquist plot at the high-frequency range (lower left-hand corner of part A). (C) Capacitance (*C*_P_) vs reciprocal of thickness (1/*r*) of single, double, and triple bR bilayers with their linear fit (black solid line). Here, the error bars show the standard variations of ∼10 junctions for each type. (D) Impedance phase (θ) vs frequency for the three different bR bilayers. The colored dots in (A) and (D) indicate the actual data, and the black solid lines fit to these data, using the equivalent circuits shown in (A).

In the vacuum environment that we use for our electrical transport measurements, the impedance Nyquist plot (Figure 9A) of the protein junctions (for all bR bilayers) contains only one, nearly perfect, semicircle, indicating a single dielectric relaxation in the junctions under these conditions. No (*R*–*C*) contribution that might be associated with the ∼0.5 to 1.0 nm SiO_*x*_ layer (regrown controllably on the p^++^-Si substrate/electrode) inside the bR junctions was detected in the experimental frequency range (10 Hz–1 MHz) that we could use for reproducible, stable data collection. The typical relaxation time, the product of the resistivity and the dielectric constant, of SiO_*x*_ is >10^3^ s,^62^ which corresponds to the frequency in mHz, where no reliable experimental data could be obtained. This Nyquist plot (under vacuum condition) fits well to an equivalent circuit of a parallel resistor–capacitor (*R*–*C*) part, with a series resistance, as shown in the right-side inset of Figure 9A. Here, the added series resistance (*R*_S_) can be assigned to an Ohmic contact resistor. However, when the experiments are done under high humidity conditions (at ambient pressure), the Nyquist plot exhibits two semicircles (Figure 9A), which can be seen by zooming in on the high-frequency range of (the lower left-hand corner of) the Nyquist plot, as shown in Figure 9B. The small semicircle, at high frequencies (Figure 9B), fits well to an *R*–*C* equivalent circuit (*R*_2_ and *C*_2_) and probably can be assigned to electronic conduction and capacitance, while the large arc (Figure 9A), at low frequencies, has a “tail” typical for proton conduction.^63,64^ This type of arc can be described by an equivalent circuit^65,66^ composed of a resistor (*R*_1_) in series to a Warburg (diffusion) element (*W*_1_) that is parallel to a capacitor (*C*_1_), as shown in the left-side inset to Figure 9A. The added series diffusion element (*W*_S_) may be attributed to ionic conduction at the contact interfaces (fitting parameters are given in Figure S14A).

#### 3.9.1 Are Electronic Charge Carriers the Only Ones?

To determine the nature of the electrical charge carriers, we measured impedance spectroscopy (IS) on a single bilayer junction. Because the large top electrode (Au pad) that completes the protein junction was deposited by floating it onto the protein film from water (see the Experimental Section), water may be trapped between the Au pad and the protein, notwithstanding extensive exposure to vacuum of the junction, before measurement. In principle, the presence of water could make embedded buffer ions mobile. Given that the biological function of bR is (photoinduced) proton pumping, proton migration across the complete junction can, thus, not be excluded. IS can distinguish qualitatively between ion and electron transport by comparing different equivalent circuits, with and without elements for proton conduction, as fitting the data to these. For our measurements in a vacuum, we find that a simple parallel *R*–*C* circuit with an added series resistance (Figure 9A) provides a very good fit (for fitting parameters see Table S3 in Figure S14B). Such a circuit does not include a Warburg element, which implies that there is no experimental evidence of ion diffusion in the dry bR junctions.

To check this interpretation, we repeated the measurements in a high-humidity environment, where ion diffusion can occur in the proteins.^67^ In >95% RH, the Nyquist plot contains an arc with a “tail” at low frequencies (Figure 9A), which fits well with a proton conduction equivalent circuit. This suggests that some *proton* diffusion takes place in the bR junctions at high humidity. Such a behavior was found with thicker BSA multilayers under a similar humidity condition.^67^ This result is important as it shows that if ion diffusion occurs, we can and will measure it via IS. As a further control we then redried the bR junction and found that the nature of IS reverts to that characteristic of the dry protein state, with a junction resistance that is about twice that of the initial dry junction before its exposure to high humidity (Figure S14B). The increase in resistance could be associated with the humidity-induced oxide growth.^68^ Therefore, we conclude that under our (10^–5^–10^–6^ mbar) vacuum measurement conditions, only electronic charge carrier transport is observed.

#### 3.9.2 Further Interpretation of Junction Capacitance Data

The interpretation of the IS measured on the different bR bilayer junctions (in vacuum), using the equivalent circuit shown in the inset (right side) of Figure 9A, is that of a combined protein resistance (*R*_P_) and a parallel protein junction capacitance (*C*_P_). We can use that capacitive behavior to explain the quality of the bR junctions for ETp measurements. *C*_P_ values were extracted from the fits of impedance data with the earlier mentioned equivalent circuit under a vacuum for the bR bilayer junctions. The *C*_P_ values vary linearly with the reciprocal thickness (*r*) of the bR bilayers (Figure 9C). The linearity of the *C*_P_ vs 1/*r* plot directly reflects the effective electrode separation increase because of adding more bR bilayers between the electrodes, as expected for a simple parallel plate capacitor. In such a capacitor, the bR bilayers are the dielectric medium, with a dielectric constant (κ)
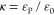
4where ε_P_ and ε_0_ are the protein layer and vacuum permittivities, and *r* is the separation between two conductive electrodes. If the electrodes have a common area *A* (that will be the area of the smallest of the two electrodes), then the junction capacitance will be
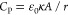
5Here the ellipsometry and AFM scratching derived protein layer thickness (Table 1) were considered as the junction length (*r*). In the *C*_*P*_ vs 1/*r* plot, only three data sets follow the typical linear trend with a high regression coefficient (*R*^2^ = 0.98). In our junction configuration, the impedance measurement was limited by the junction length. The thicker the protein junction, the more capacitive it becomes, which directly reflects in the phase at the low-frequency (Figure 9D) region. As all the impedance measurements were done with minimal AC bias (10 mV; with 0 DC bias; see the Experimental Section) sufficiently conductive junctions are needed in order to get a measurable impedance signal, and the poor conductivity of thicker layers (above the triple bilayer) did not allow impedance measurements. From the slope the estimated κ is ∼5.5 (Figure 9C), by substituting the known geometric junction area (∼2 × 10^–3^ cm^2^) of the top contact. The slight deviation from a straight line indicates that κ decreases upon the addition of bilayers.

Figure 9C is the first estimate of the dielectric constant derived from direct IS experiments for a solid-state dry protein film. In the literature, values were derived mostly from computational studies. The estimated κ value of bR bilayer films is in reasonable agreement with reports on other proteins that yielded κ values of 6–7^53^ or even 3.5–10.^69^ In another study of solid-state thin protein films, the average κ was ∼3.3^70^ for the cofactor-free proteins, and the inclusion of an organic or inorganic cofactor led the κ to increase to 6–7.

#### 3.9.3 Impedance Phase Plot

By comparing the phase plots for the junctions with different bR bilayers (under vacuum conditions), one can evaluate the quality of the junctions. In Figure 9D, the phase (θ) reaches 90° at high frequencies, irrespective of bilayer thickness, which suggests a full capacitive behavior of the bR junctions at high frequency. This result is consistent with the behavior typical for a parallel *R*–*C* circuit (here the bR junction): at low frequencies, it behaves as a complete resistor (θ = 0 or small positive) and as a complete (full) capacitor (θ = 90°) at high frequencies.^71^ The full capacitive behavior of bR junctions suggests an insignificant contribution of pinholes. Ergo, ETp occurs mostly via bR proteins rather than via pinholes.

### 3.10 ETp Mechanism

Recently, Gupta et al.^45^ reported a low *β* (0.28 nm^–1^) and activationless ETp in a ferritin system, interpreted as electron tunneling over the 7.5 nm polypeptide matrix-incorporated FeO(OH) junction. Interestingly, 7.5 nm is beyond the 7 nm limit of quantum mechanical tunneling through multiheme proteins, derived by Futera et al.^44^ The latter, theoretical analyses of experimentally observed currents via a multiheme cytochrome protein led to the conclusion that the transport mechanism will change from coherent tunneling to incoherent hopping for ETp over >∼7 nm protein distances. That conclusion holds in these highly electron-rich proteins if ETp is not limited by interfacial ET between the electrode and the protein. However, we note that unlike the multiheme proteins, bR lacks obvious hopping sites (and is less electron-rich).

Analysis indicated weak exponential voltage-dependent NDC at low bias and therefore excluded hopping as a possible transport mechanism (cf. Section 3.7). The temperature-dependent *I*–*V* results yield low to negligible transport activation energies, i.e., barriers for transport, in terms of the thermal energy, which is a necessary, but not a sufficient, finding as support for the operation of a tunneling mechanism. At low applied bias conditions, any tunneling is expected to be nonresonant as the electrode’s *E*_F_ is unlikely to be near a protein frontier orbital energy level (Figure 6). Only in organic molecules with extended conjugation that were measured, with strongly reduced HOMO–LUMO gap, can the electrode Fermi level (*E*_F_) be energetically close to accessible molecular energy levels, which might assist electron hopping or lead to resonant tunneling.^51^ However, the wide HOMO–LUMO gap in bR bilayers makes such a scenario unlikely.

Near temperature-independent currents, with a weak voltage dependence (NDC < 3), point to non-resonant electron transport due to lack of overlap between the energy levels of the protein and the electrode *E*_F_ (Figure 6). Moreover, such kind of transport is solely incoherent in nature simply by considering charging/discharging at the junction interfaces and dephasing due to unpopulated protein energy levels near *E*_F_.^72^ In addition, weakly coupled (noncovalently bonded) protein–electrode junctions^72^ also support such incoherence within the Landauer model.

We consider operation of the quantum mechanical tunneling as extremely improbable because of the expected decay of the wave function, i.e., decrease of electron density probability with distance from injection locus across the protein. If we still try to fit the low-voltage current data to a non-resonant tunneling model, such as the Simmons one, we find that only the SiO_*x*_ and linker-coated SiO_*x*_ fit well to a non-resonant tunneling model, while the results for protein junctions strongly deviate from such model (SI Section 5, Figure S15).

One possible way to reconcile these results with existing physical models is to assume that the rate-limiting step for transport is electron injection across the contact (see Section 3.8) that is least efficient (for charge injection/extraction) and that once an electron is injected into/extracted from the protein to/from the electrode, it will reach the other electrode and will be extracted from/injected into the other electrode, with little further decay. A model of Papp and Vattay et al.^73^ predicts such behavior. However, what could be the charge injection/extraction mechanism remains a riddle and awaits further work.

At the same time, the lack of significant temperature dependence of ETp and, thus, of thermal activation energy (Section 3.3) suggests that the protein energetics, including those at the interfaces with the contacts, do not change significantly with increasing protein layer width. There is, however, a small but significant variation of bR ETp with junction length (Figure 4A), which seems at odds with ETp that is dominated by the protein–electrode contact.

We note that if indeed the dielectric constant decreases with increasing protein layer width (cf. Section 3.9.2), the efficacy of electrostatic screening of charges injected in the protein will decrease. As a result, the charge balance at the protein–electrode contact will change. This will affect the rate at which charges can be injected/extracted, which will translate to decreased currents at a given voltage. Another factor that can affect the contact–protein charge balance is that of the internal interface charges. The reason is not just the added number of interfaces but also that films with >1 bilayer contain new interface types. The internal bR/bR interface within the bilayer is that of cytoplasmic/cytoplasmic sides, while the interface between bilayers is extracellular/extracellular, with likely amide bonding due to the EDS treatment. However, both types of bR surfaces are negatively charged, and it is conceivable that the interactions between the layers induce protein charge reorganization. Such reorganization, which can cause slight orientation changes of charged groups and/or of the countercharges that will deposit together with the protein layers, will also be reflected in the electrical charges at the contact (electrode–protein) interface. Any increase in local negative charge density in/at the protein/electrode contact will reduce the rate of electron injection into/extraction from the protein.

In addition, the experimentally measured increase in the roughness of bR films with increasing film width (Figures S1, S5, and S6) implies a decrease in the actual area that is electrically active (involved in current transport) between the protein and the top contact.

All these factors may be involved in the orders of magnitude decrease in current observed with an increasing bR film width.

## 4 Conclusions

In this work, we showed that protein junctions, whose quality was assessed by electrical means (impedance and breakdown), exhibit unique long-range charge transport ability (*β* < 0.6 nm^–1^) over up to ∼60 nm junction lengths. The actual transport activation energy (by subtracting the effect of the high-doped Si substrate) is found to be exceptionally low (<*k*_B_*T*) for all bR junctions, irrespective of their thickness. Even under a small applied bias (a few tens of mV), these protein junctions allow efficient electronic transport. This result is the more surprising one because of the large (tens of *k*_B_*T*) energy separation between the nearest protein energy and electrode Fermi level (measured before top contact deposition). Therefore, such protein–electrode energetics and near-activationless transport do not fit with the operation of a hopping-type mechanism. The low transport activation energy and low exponential voltage dependence could point to quantum mechanical tunneling. Still, such an explanation is not plausible because the lengths involved are way above those for which tunneling currents can be measured through what is basically a disordered insulating medium. None of the several common transport models for electron transport across proteins (and nonconjugated molecules in general) that we tested by trying to fit our observed currents as a function of length, voltage, and temperature appear to be applicable. Recently, Li et al.^74^ reported electron tunneling with a coherence signature for <2.0 nm alkane dithiol junctions. Except for the roughly *T*-independent ETp, we do not have a way to measure coherence in our, or for that matter any, protein junctions. Possibly quantum mechanical tunneling reflects transport through one of the contacts, which still leaves the question of how the electrons pass through up to 60.0 nm of bacteriorhodopsin multilayers. The overall ETp behavior of our protein junction suggests a type of transport mechanism that is beyond the established conventional mechanisms.

## Supporting Information Available

Discussion of AFM scratching and image processing, MatLab-based current–voltage data processing, high-humidity setup for impedance measurement, HOMO–LUMO gap of bR, and the non-resonant tunneling model fitting (PDF)
