# Supplementary material for: Near-Temperature-Independent Electron Transport Well beyond Expected Quantum Tunneling Range via Bacteriorhodopsin Multilayers
Source: J Am Chem Soc. 2023 Nov 7;145(45):24820–35. doi: 10.1021/jacs.3c09120 (PMC10655127; doi:10.1021/jacs.3c09120)
Supplement: Supplementary file 1 — ja3c09120_si_001.pdf [file ja3c09120_si_001.pdf]

Supporting Information

for

Near-Temperature-Independent Electron Transport Well beyond Expected Quantum Tunneling Range via Bacteriorhodopsin Multilayers

Sudipta Bera,[*,†] Jerry A. Fereiro,[†,&] Shailendra K. Saxena,[†,@] Domenikos Chryssikos,[‡,§] Koushik Majhi,[†] Tatyana Bendikov,[∥] Lior Sepunaru,[⊥] David Ehre,[†] Marc Tornow,[‡,§] Israel Pecht,[#] Ayelet Vilan,[*,%] Mordechai Sheves,[*,†] and David Cahen[*,†]

[†]Department of Molecular Chemistry and Materials Science, Weizmann Institute of Science, Rehovot 7610001, Israel
[‡]Molecular Electronics, Technical University of Munich, 85748 Garching, Germany
[§]Fraunhofer Institute for Electronic Microsystems and Solid State Technologies (EMFT), 80686 München, Germany
[∥]Department of Chemical Research Support, Weizmann Institute of Science, Rehovot 7610001, Israel
[⊥]Department of Chemistry and Biochemistry, University of California, Santa Barbara, California 93106, United States
[#]Department of Immunology and Regenerative Biology, Weizmann Institute of Science, Rehovot 7610001, Israel
[%]Department of Chemical and Biological Physics Weizmann Institute of Science, Rehovot 7610001, Israel
[&]School of Chemistry, Indian Institute of Science Education and Research, Thiruvananthapuram 695551, Kerala, India
[@]Department of Physics and Nanotechnology, College of Engineering and Technology, SRM Institute of Science and Technology, Kattankulathur, Chennai 603203, Tamil Nadu, India

*Email david.cahen@weizmann.ac.il

*Email mudi.sheves@weizmann.ac.il

*Email ayelet.vilan@weizmann.ac.il

*Email sudipta.bera@weizmann.ac.il

1. Insight in AFM Scratching

The AFM scratching results of the T-60 bR multilayer (Figure S1B) shows incomplete removal of bR molecules at the scratched area, despite of the high applied contact force (~200 nN). Due to the incomplete removal of bR molecules, the estimated depth profile (Figure S1C) shows reduced thickness compared to the thickness (60 nm) deduced from ellipsometry.

To derive the overall layer thickness, we used a technique of AFM-scratched image analysis based on color-mapped height profiles (Figures S4A-D) for different bR multiple bilayers, where the minimum height of exposed protein layer was set to be zero (by zero correction using *Gwyddion 2.63*). The height corresponding to the color of the unscratched region, shows the layer thickness associated with the color bar. In Figures S4A-D analysis, green represents the average thickness of each bilayer, which nicely varies from one layer to another. In addition, area-based histogram analysis was also done on the scratched images, which essentially showed two height peaks (Figure S4E). One peak corresponds to the average lowest-height exposed area (scratched region) and the other one is the average height of the unscratched region. Therefore, the difference of the height peaks, directly gives the layer thickness.

2. MatLab Based Data Processing

First, raw data (V, I) were fitted (fit.m) to a spline function using: smF = fit(V,I, ‘smoothingspline’, ‘SmoothingParam’,0.999995). Then, the smoothed current is smI = feval(smF, V) and the direct (‘linear’) derivative was computed as G = differentiate(smF, V). Finally NDC = G.*V./smI.[1]

3. Setup to Maintain High Humidity Environment for Impedance Measurement

The protein junction was kept in a humidity chamber for 24h, which results in the appearance of several tiny droplets over the substrate (> 95% RH). Then, we transferred the humidity treated junction in the Lakeshore chamber and measured the impedance immediately. Before transferring the sample to the Lakeshore chamber (in probe station), we put a small Petri dish with wet filter paper and kept it for few hours with closed

chamber lid. In this setup we maintained the high humidity; here the tiny droplet acts as an indicator that persists during the measurement. Moreover, without nitrogen drying, such protein junction cannot be recovered to dry protein junction characteristic (semicircular Nyquist plot) even after 3-4 h.

4. HOMO-LUMO Gap of bR

The allowed energy levels of bR were derived from the UV-vis of bR. The UV-vis absorption shows the characteristic absorption peaks ~280 nm and ~578 nm due to the aromatic amino acid residues (of the polypeptide) and the retinal part of bR, respectively. The absorption edge at the longest wavelength (~650 nm) corresponds to the HOMO-LUMO gap of bR with equivalent energy ~1.9 eV. The shorter wavelength absorption edge (~460 nm) of the retinal corresponds to the energy gap of 2.7 eV relative to the HOMO. The nearly-forbidden energy gap of ~1.3 eV lies between the longer wavelength absorption edge (~310 nm; 4.0 eV) of polypeptide energy levels (violet line in Figure S10 right) and the shorter absorption edge of the retinal.

5. Fitting with Non-Resonant Tunneling Model

Simmons tunneling under low bias (V → 0 Volt)[2]

$$I \propto V \exp\left(-\frac{2r\sqrt{2m_e\phi}}{\hbar}\right) \qquad [S1]$$

$$\ln(I) = \ln(V) - \frac{2r\sqrt{2m_e\phi}}{\hbar} + constant \qquad [S1a]$$

Here, I is the junction current under the applied bias, V; r is the protein layer thickness, ϕ is the energy height of the barrier, m_e the mass of the electron, and $\hbar = h/2\pi$, is the reduced Planck constant.

The equation S1a says ln (I) should vary linearly with ln (V) with a slope (= 1) in the low bias regime (V → 0). The experimental I-V curves closely fit for the junctions of just silicon oxide and linker-coated silicon oxide with a slope (~1.1). On the contrary, the fitted slopes largely deviate from 1 for every bR bilayer junction. Interestingly, for all bR bilayers the slopes are nearly identical (~0.1) (Figure S15), irrespective of protein-layer thickness.

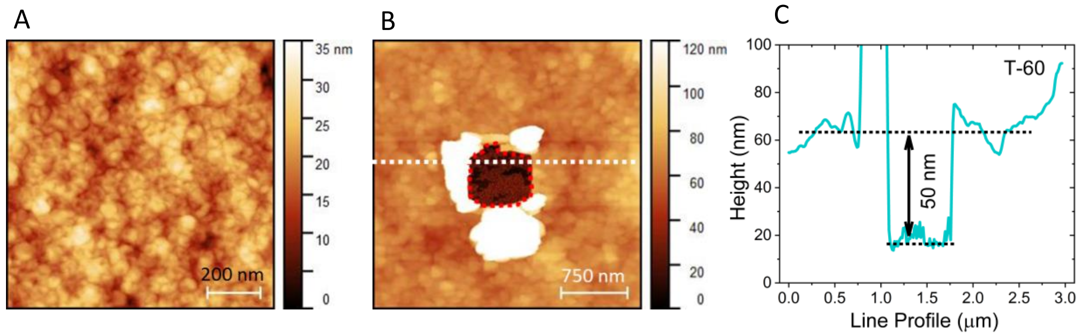

Figure S1: (A) Tapping mode AFM topography of 60 nm (ellipsometry) thick bR multilayer (T-60) on top of the linker-coated silicon (rms roughness ~5 nm). **(B)** Topography of tapping mode AFM image over scratched area of T-60 bR layer **(C)** Depth profile (white dotted line shown in **(B)** at the scratched region with the thickness ~50 nm.

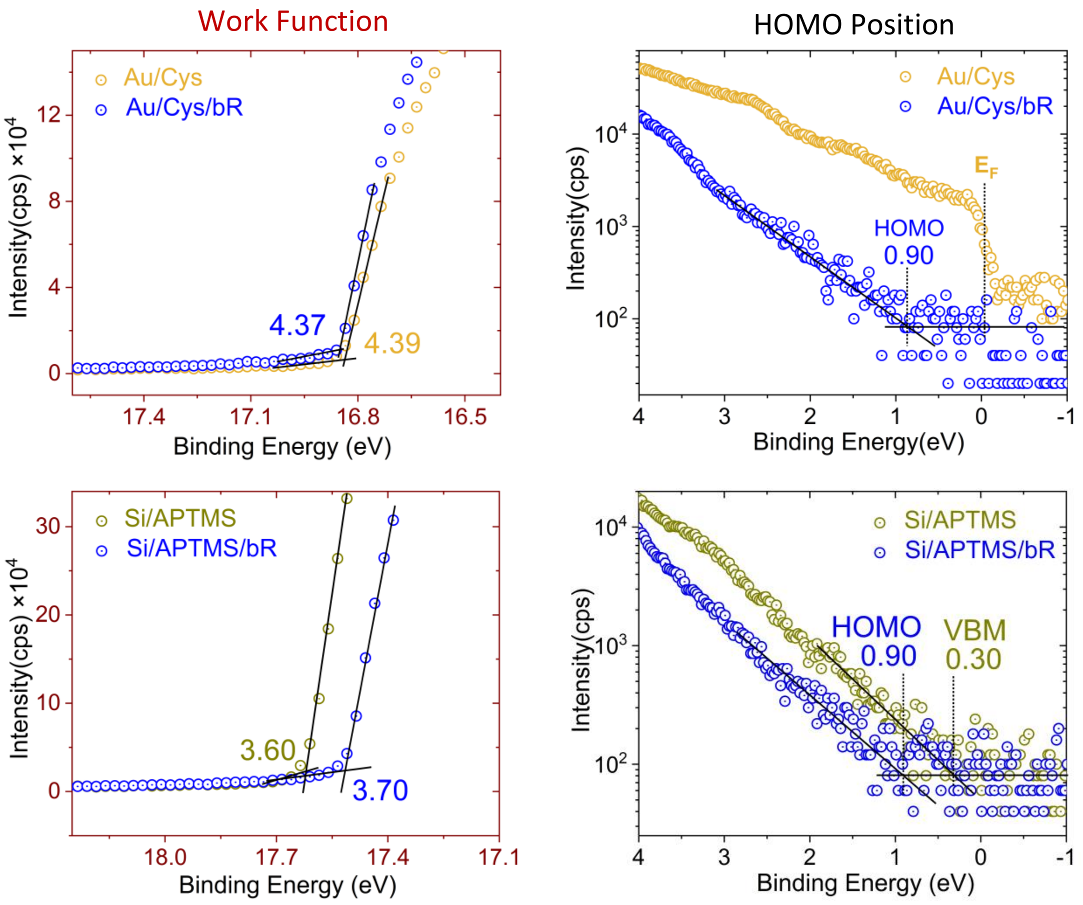

Figure S2: Secondary electron photoemission intensity as function of binding energy (from the photon kinetic energy and the work function, for Cysteamine (Cys)-coated Au, APTMS-coated Si (p^{++}), and bR single bilayer on top of linker-coated substrate, as noted in the respective figures. **LEFT:** Extraction of work function values and those values from the secondary electron cut-off (SECO) energies. **RIGHT:** same for HOMO positions near the Fermi level, where the photoemission signal is plotted on a log scale, because of the low density of states in the proteins. **TOP:** the Au Fermi edge is indicated; **BOTTOM:** the VBM of the Si is indicated.

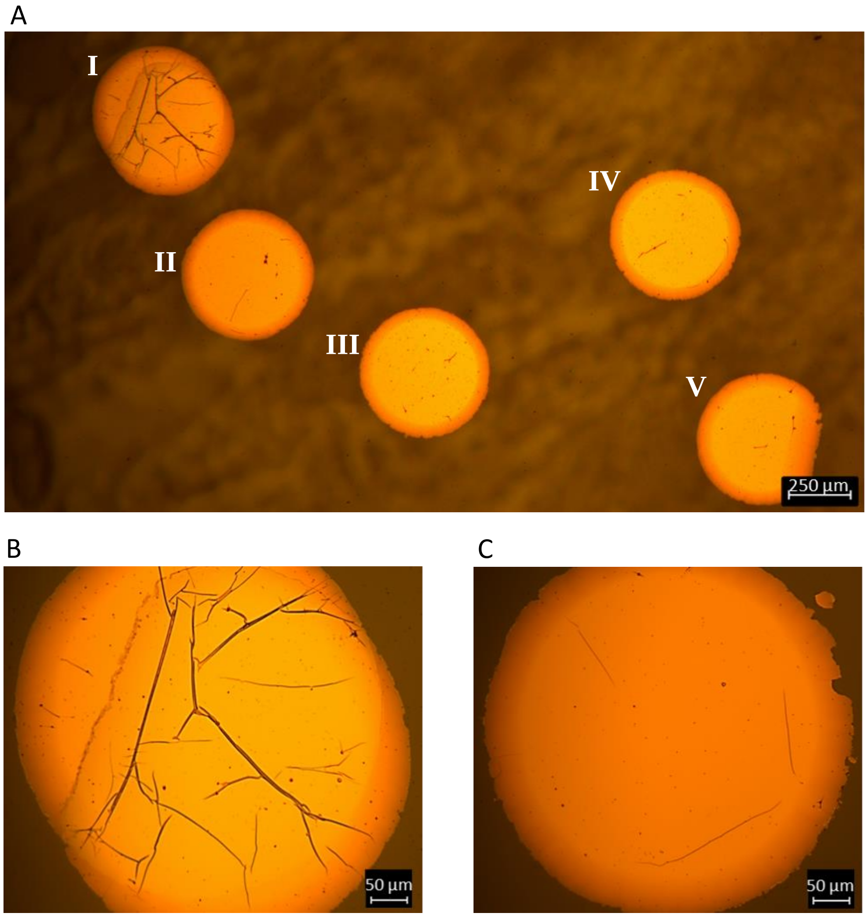

Figure S3: White light photographs of **Top-electrode** (~500 µm diameter Au-pads; LOFO) on protein layer, using optical microscope. **(A)** A set of five LOFOs; we selected complete round shaped (unlike pad **V**), wrinkle-free (unlike **I**) flat LOFO surfaces (like **II**, **III**, and **IV**) as a top electrodes for ETp measurements. **(B)** Zoomed image of surface with wrinkles (discarded for the measurement), and **(C)** close to wrinkle-free surface of a Au-pad, which is the type used for our measurements.

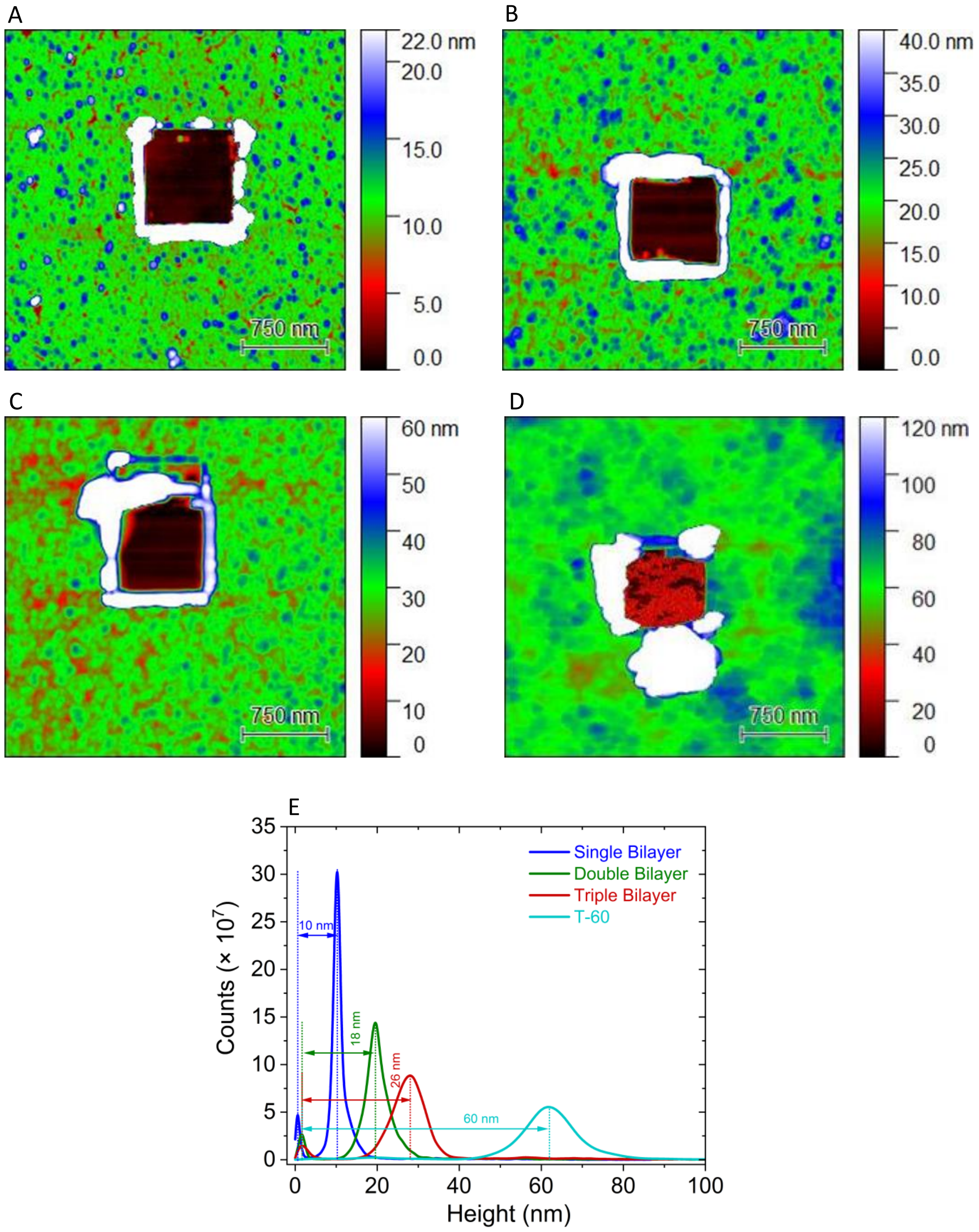

Figure S4: Rainbow-colored AFM scratched images of **(A)** Single Bilayer, **(B)** Double Bilayer, **(C)** Triple Bilayer and **(D)** T-60 bR multilayer. Here the black area is the minimum exposed area considered to be zero height and the green shows the average thickness of each protein layer. **(E)** Shows the area profiles of scratched images with Gaussian fit of height histograms (generated by *Gwyddion 2.63*) for the scratched images of **A-D**, where each layer thickness was estimated from the height gap between high and low intensity peaks (see **SI** section **1**).

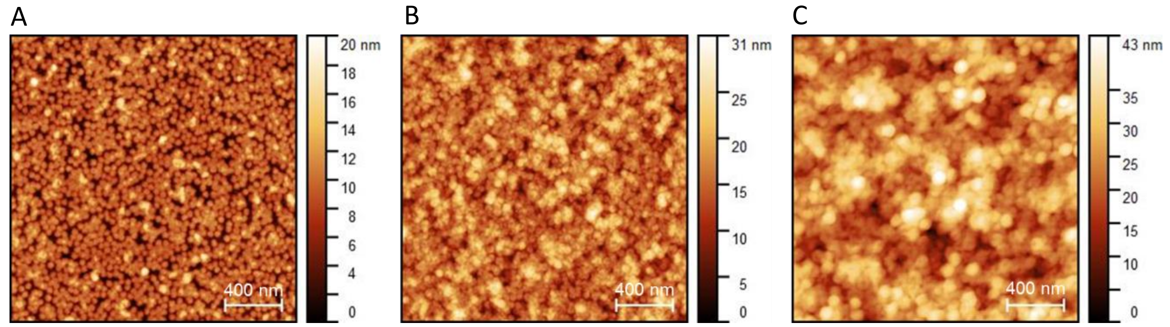

Figure S5: Tapping mode AFM topography of bR bilayers on APTMS-coated SiOx/Si; **(A)** Single Bilayer (rms roughness 2.2 ± 0.3 nm), (**B**) Double Bilayer (rms roughness 3.1 ± 0.2 nm), and **(C)** Triple Bilayer (rms roughness 4.3 ± 0.5 nm).

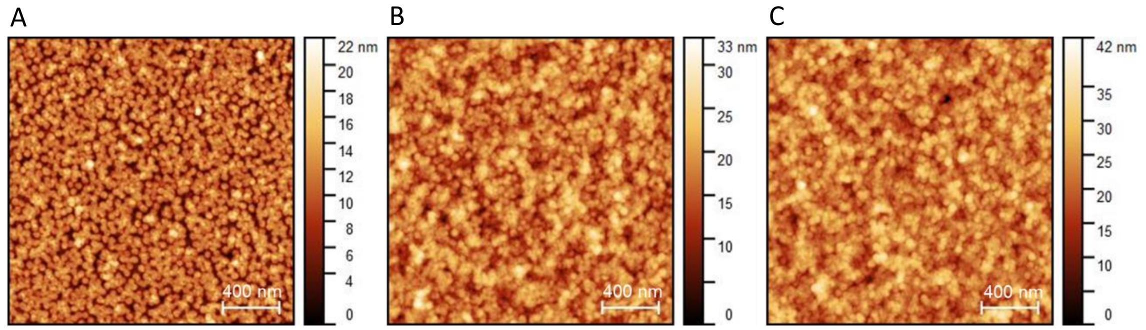

Figure S6: Tapping mode AFM topography of bR bilayers on cysteamine-coated Au; **(A)** Single Bilayer (rms roughness 2.3 ± 0.2 nm), **(B)** Double Bilayer (rms roughness 3.0 ± 0.3 nm), and **(C)** Triple Bilayer (rms roughness 3.6 ± 0.3 nm).

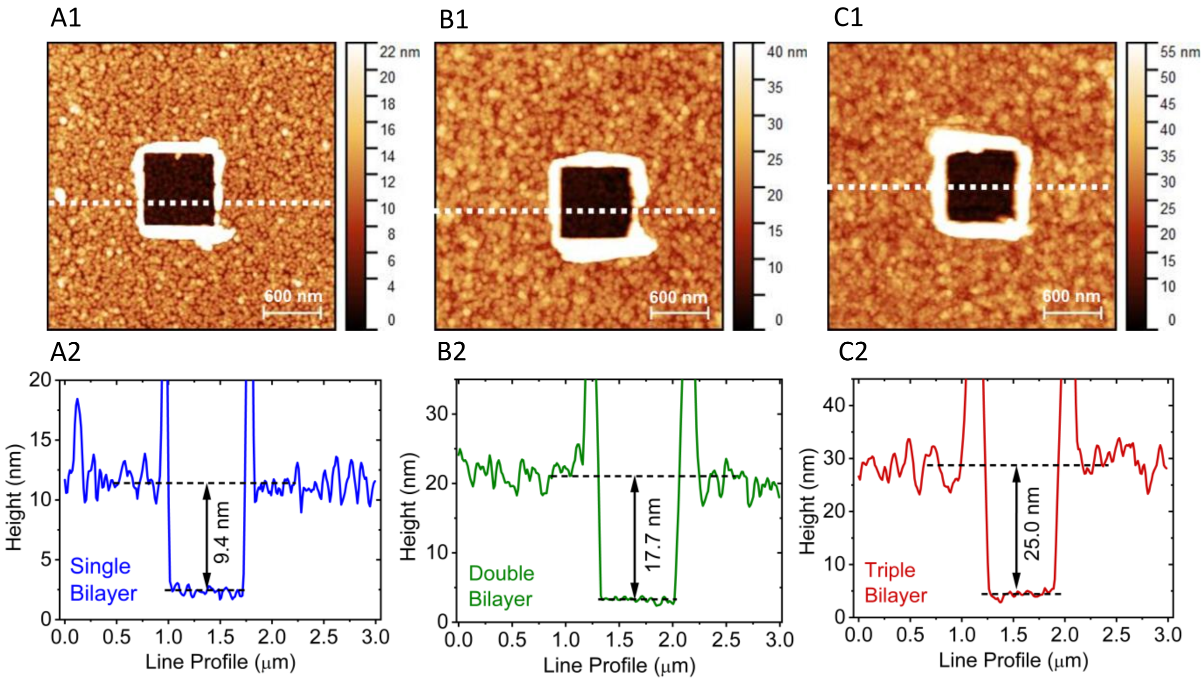

Figure S7: AFM scratching images of different bR bilayers on cysteamine-coated Au; **A1**, **A2** for Single Bilayer; **B1**, **B2** for Double Bilayer; **C1**, **C2** for Triple Bilayer. **Top row** shows the scratched image topographies for the different bR bilayers. The white dotted lines indicate where the line profiles (**bottom row**) were measured. The profiles show the depths of the scratched regions for each of the samples in the top row.

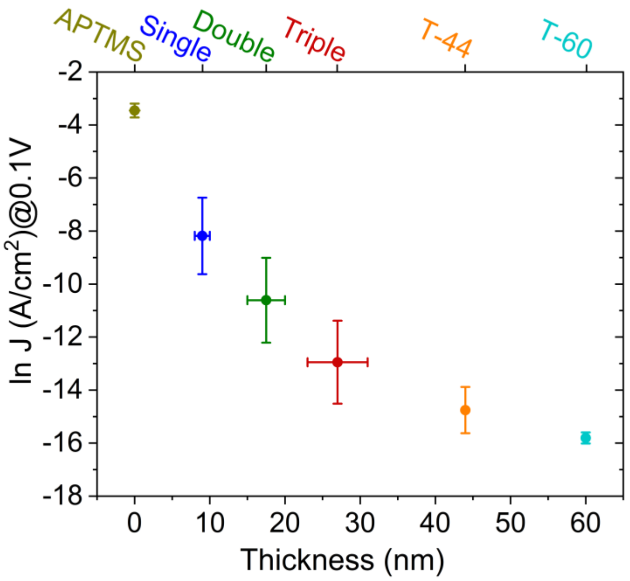

Figure S8: Plot of ln *J* vs. thickness (*r*) for the five different protein films used in this study. The junction configurations are p^{++}-Si/SiO_x / APTMS / bR / Au. All currents are obtained with +0.1 V applied bias at RT (293 ± 2 K). The differential slope (d ln*J*/d*r*) extracts the *β* values (see inset **Figure 4C**) for different bR bilayers.

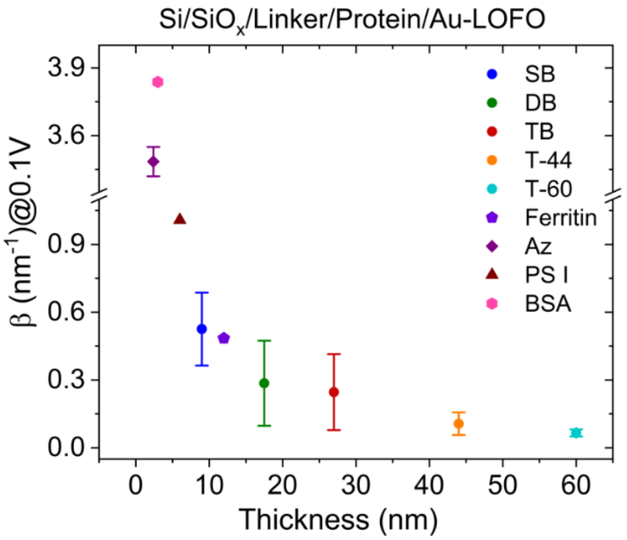

Figure S9: A plot of length decay constant (*β* value @ 0.1 V) against the thickness of mono (or, for bR, bi-)layers of different proteins, including all bR multilayer junctions. The data were obtained with the same p^{++}-Si/SiO_x/Linker/Protein/Au-LOFO junction configurations. Ferritin,[3] PS I,[3] Az,[4,5] and BSA[4] data are taken from our previous work.

Table S1A				
**Protein Layer**	**E_a@-50 mV**		**E_a@-100 mV**	
	Near RT	< 160K	Near RT	< 160K
Single Bilayer	6.0 ± 1.0	1.0 ± 0.05	13.0 ± 1.0	1.5 ± 0.20
Double Bilayer	7.0 ± 0.5	1.5 ± 0.1	7.0 ± 0.5	1.5 ± 0.20
Triple Bilayer	10.0 ± 1.0	1.5 ± 0.30	8.0 ± 1.0	0.10± 0.10
T-60	23.0 ± 1.5	1.5 ± 0.50	23.0 ± 1.0	3.0 ± 1.00

Table S1B				
**Protein Layer**	**E_a@50 mV**		**E_a@100 mV**	
	Near RT	< 160K	Near RT	< 160K
Single Bilayer	16.0 ± 2.0	0.6 ± 0.10	18.0 ± 1.0	0.4 ± 0.05
Double Bilayer	18.0 ± 1.0	2.0 ± 0.20	17.0 ± 0.5	0.20 ± 0.10
Triple Bilayer	16.0 ± 1.5	1.6 ± 0.30	15.0 ± 1.0	0.80 ± 0.10
T-60	32.0 ± 3.0	0.4 ± 0.30	27.0 ± 1.5	0.10 ± 0.05

Table S1: Activation energies (E_a) for thermal activation of currents across p^{++}-Si/SiO_x/APTMS/bR/Au junctions with different bR-bilayers at ± 50 mV and ± 100 mV, applied bias both near RT and at low temperatures (<160K). **TOP** table (**1A**) for E_a estimated at negative bias and **BOTTOM** table (**1B**) for positive bias.

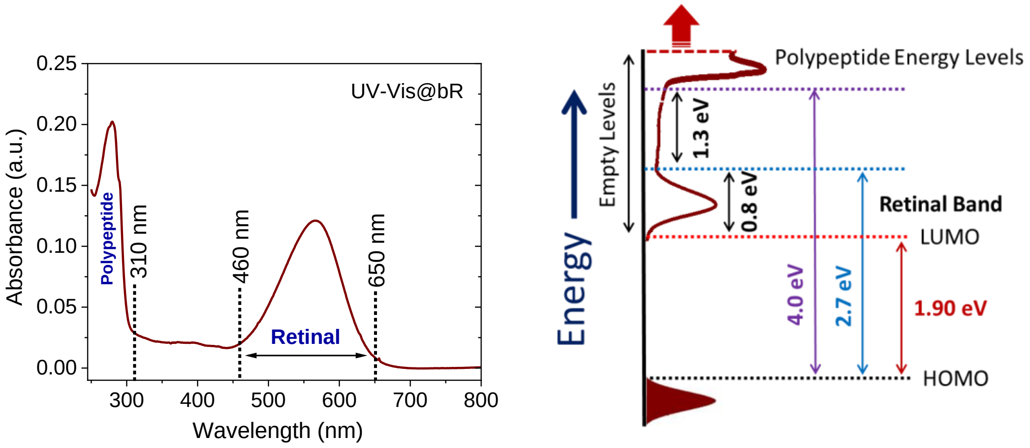

Figure S10: UV-vis absorption spectra (1cm path length) of 4μM bR in AS-PB buffer (**LEFT**) and UV-vis derived protein-energy levels with HOMO-LUMO gap (**RIGHT**).

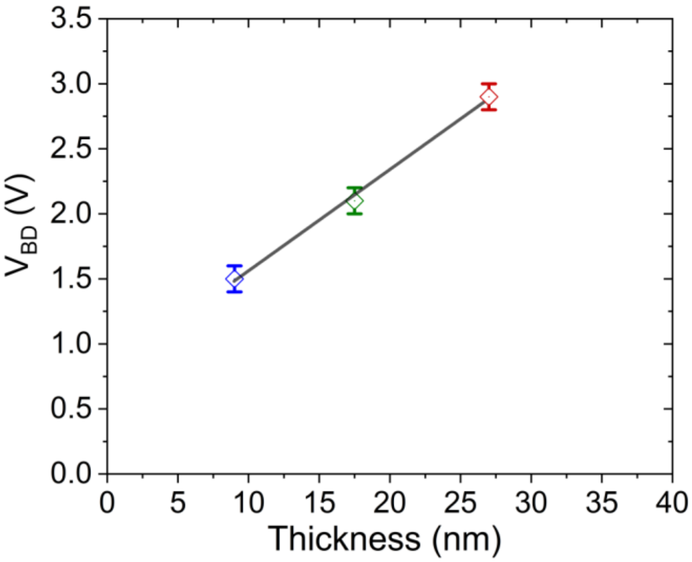

Figure S11: A plot of voltage breakdown with the protein layer thickness for **Single** (*blue*), **Double** (*green*), and **Triple** (*red*) bilayer junction, shows a constant electric field ~0.1 GV/m, which is required for electrical breakdown of different bR junctions.

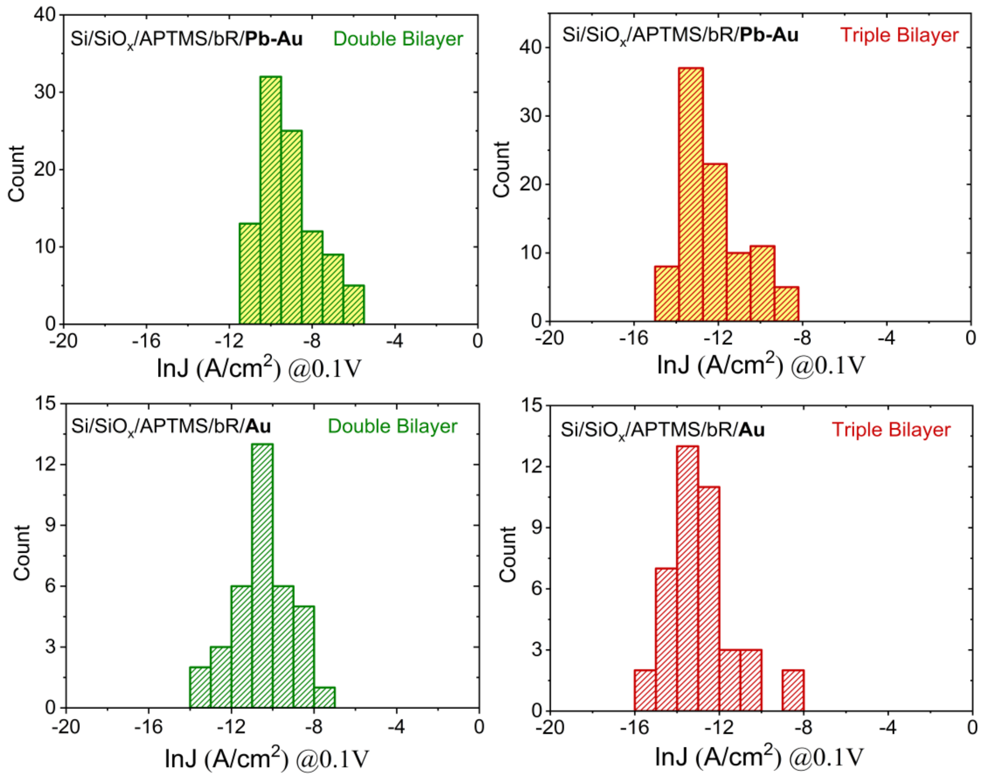

Figure S12A: The junction current density histogram (@0.1 V) of bR **double bilayer** junction (*left column*) and **triple bilayer** (*right column*) with different top electrode contacts; Pb-Au (***top-row***) and Au-LOFO (***bottom-row***).

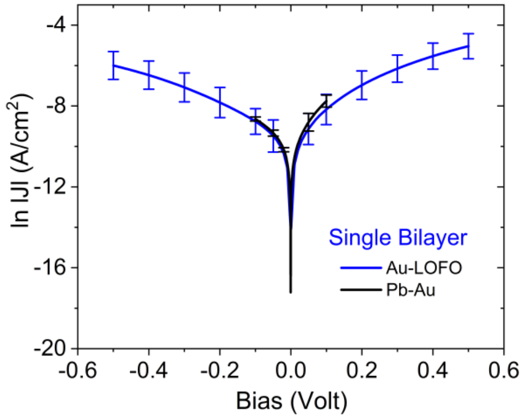

Figure S12B: ln *J* vs. *V* plot of single bilayer bR junction on APTMS coated Si/SiO_x with two different top electrodes at RT. The ***blue line*** represents data for the mechanically deposited Au-LOFO top electrode, for single bilayer junctions (± 0.5 V bias range), and the ***black line*** for thermally evaporated Pb-Au top electrodes; averaged over5 junctions (± 0.1 V bias range).

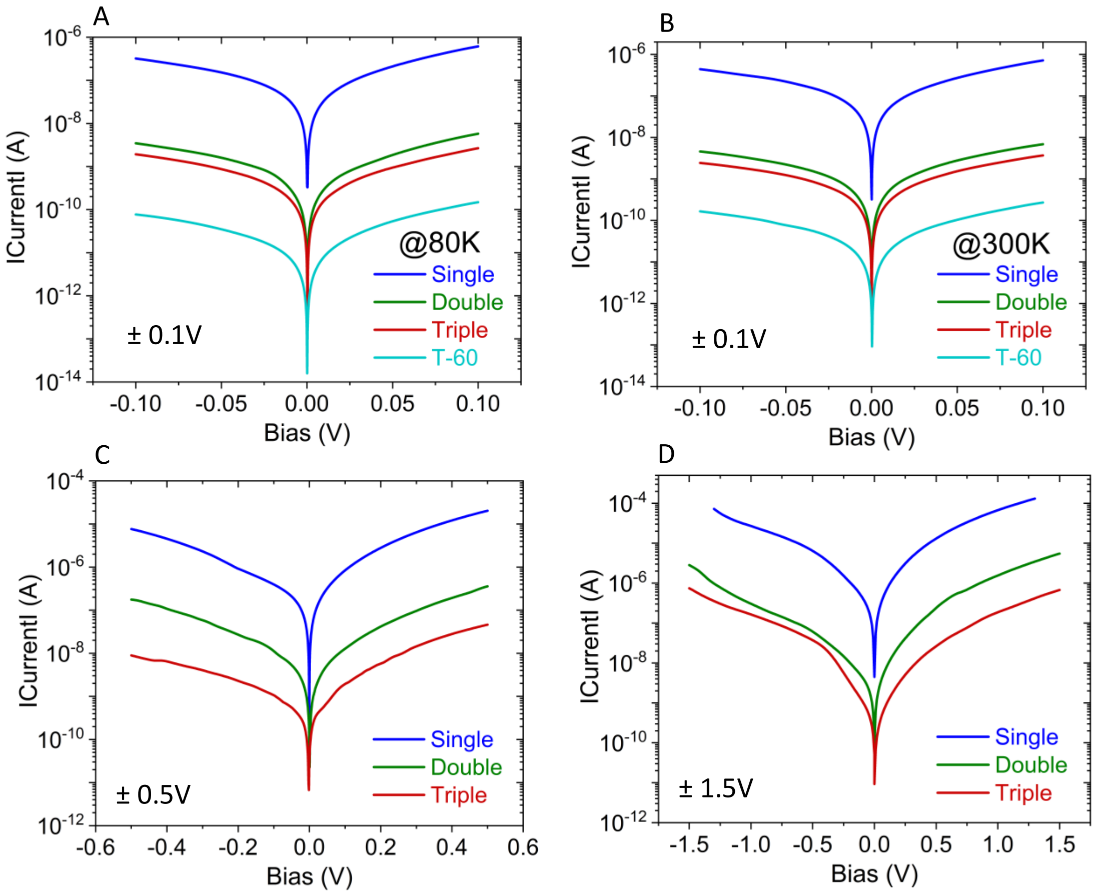

Figure S13: Current-voltage (semi-log) response (used in NDC plot) of different bR bilayers including T-60 under the different applied bias and temperature conditions. **(A)** ± 0.1 V at 80K, **(B)** ± 0.1 V at 300K, **(C)** ± 0.5 V at RT, and **(D)** ±1.5 V at RT for double and triple bilayer, ± 1.3 V for single bilayer to avoid destroying the junction (voltage breakdown). No low temperature data was taken at the high voltage sweep as the junctions were not stable.

Equivalent Circuit under High Humidity

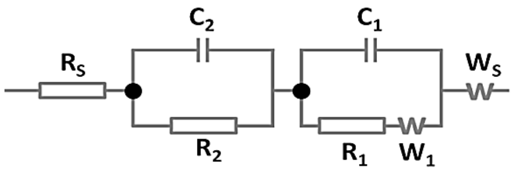

Table S2: Impedance fitting based estimated value of different circuit elements (χ^2 ~0.004)							
Elements	R_S (Ω.cm^2	C_2 (μF/cm^2)	R_2 (Ω.cm^2)	C_1 (μF/cm^2)	R_1 (Ω.cm^2)	W_1 (Ω.cm^2)	W_S (Ω.cm^2)
Value	0.08±0.007	1.67±0.05	4.70±0.26	2.69±0.11	23.44±0.40	115±16	197±17

Figure S14A: The equivalent circuit (***top***) elements for the impedance of bR single bilayer junction under high humidity (> 95% RH) condition with the parameter values (***bottom***) obtained for the best fit (solid line in Figure 9A) (***Table* S2**).

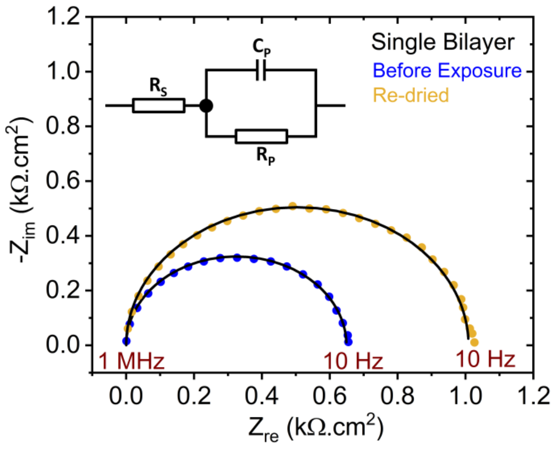

Table S3: Impedance fitting parameter		
Circuit elements	Dry	Re-dried
R_S (Ω.cm^2)	0.055 ± 0.003	0.063 ± 0.004
C_P (μF/cm^2)	0.43 ± 0.001	0.39 ± 0.001
R_P (Ω.cm^2)	647.4 ± 2.0	1008.8 ± 3.3
χ^2	0.003	0.004

Figure S14B: Nyquist plots of single bilayer bR-junction *under vacuum* **before exposure** to high humidity (*blue dots*) and for the same sample **re-dried**, i.e., after removing it from the high humidity ambient, placing it back into vacuum and re-measuring it in vacuum after 1 day (yellow dots). The dots are the real data points and the black solid lines are the fits with the equivalent circuit, shown as inset (***top-left***), the same circuit as shown in Figure 9A as right-hand side inset. The values of the parameters for the fits to the data are shown in **Table S3**.

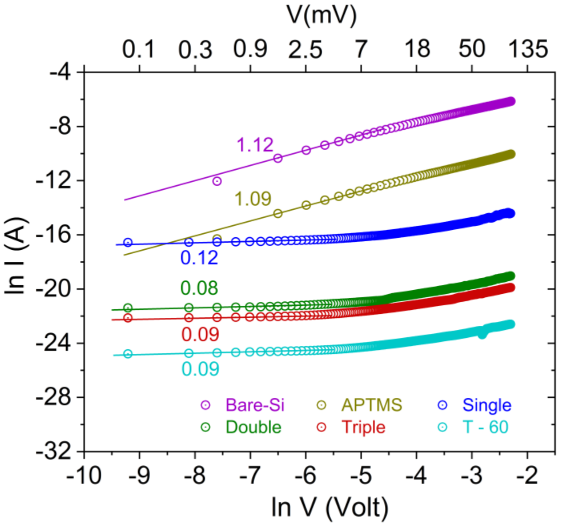

Figure S15: ln I vs. ln V plots of p^{++}-Si/SiO_x/APTMS/bR/Au junctions with different bR-bilayers (single, double, and triple), and with a ~60 nm thick bR multilayer (T-60), including the junctions (without protein) like, p^{++}-Si with < 1 nm oxide (bare Si/SiO_x) and ~0.5 nm APTMS linker bound to the Si/SiO_x. The slope near low bias ($V \rightarrow 0$), is ~1.1 without protein and ~0.1 for the different protein junctions (cf. slope values indicated at each curve).

Author contributions

S.B., D.Ca., A.V., M.S., L.S., and I.P. designed the experiments; S.B. fabricated all protein junctions, carried out all I-V and related experiments and analyzed the results for all junctions. S.B. characterized the protein layers using AFM, Ellipsometer, PMIRRAS etc. D.Ch. suggested the AFM scratching measurements and contributed, together with M.T. and J.A.F., to the interpretation of the electrical data. K.M. prepared and purified the bR protein. The UPS study was done by T.B., who analyzed the data, with S.B. L.S., D.E., and M.T. suggested the impedance measurements. S.B. and S.K.S. set up the impedance experiment setup and S.B. collected and analyzed the impedance data, with D.E. and L.S. NDC analysis was done by A.V. S.B., D.Ca., A.V., M.S., and I.P. wrote the manuscript. All the authors discussed the results and edited the manuscript.

References

(1) Vilan, A. Revealing Tunnelling Details by Normalized Differential Conductance Analysis of Transport across Molecular Junctions. *Phys. Chem. Chem. Phys.* **2017**, *19*, 27166–27172.

(2) Beebe, J. M.; Kim, B.; Gadzuk, J. W.; Daniel Frisbie, C.; Kushmerick, J. G. Transition from Direct Tunneling to Field Emission in Metal-Molecule-Metal Junctions. *Phys. Rev. Lett.* **2006**, *97*, 026801.

(3) Mukhopadhyay, S.; Karuppannan, S. K.; Guo, C.; Fereiro, J. A.; Bergren, A.; Mukundan, V.; Qiu, X.; Castañeda Ocampo, O. E.; Chen, X.; Chiechi, R. C.; McCreery, R.; Pecht, I.; Sheves, M.; Pasula, R. R.; Lim, S.; Nijhuis, C. A.; Vilan, A.; Cahen, D. Solid-State Protein Junctions: Cross-Laboratory Study Shows Preservation of Mechanism at Varying Electronic Coupling. *iScience* **2020**, *23*, 101099.

(4) Ron, I.; Sepunaru, L.; Itzhakov, S.; Belenkova, T.; Friedman, N.; Pecht, I.; Sheves, M.; Cahen, D. Proteins as Electronic Materials: Electron Transport through Solid-State Protein Monolayer Junctions. *J. Am. Chem. Soc.* **2010**, *132*, 4131–4140.

(5) Sepunaru, L.; Pecht, I.; Sheves, M.; Cahen, D. Solid-State Electron Transport across Azurin: From a Temperature-Independent to a Temperature-Activated Mechanism. *J. Am. Chem. Soc.* **2011**, *133*, 2421–2423.
